# Carbon Quantum Dots as Versatile Nanosystems for Biomedical Innovation: Mechanisms, Applications, and Translational Prospects

**DOI:** 10.3389/bjbs.2026.15960

**Published:** 2026-04-02

**Authors:** Jasdev Singh Maan, Milind Kuruvath Santhosh, Stevelyn Jia Xin Lee, Nicole Zi Yu Leow, Anis Sofia binti Mohd Adli, Charlotte Jia Qi Tai, Jia Hui Lim, Yasheni Muniandy, Chun-Wai Mai, Tong Ling Tan, Soi Moi Chye, Rhun Yian Koh, Chooi Ling Lim

**Affiliations:** 1 School of Health Sciences, IMU University, Kuala Lumpur, Malaysia; 2 Institute of Research, Development and Innovation, IMU University, Kuala Lumpur, Malaysia; 3 Department of Pharmaceutical Chemistry, School of Pharmacy, IMU University, Kuala Lumpur, Malaysia; 4 Division of Applied Biomedical Science and Biotechnology, School of Health Sciences, IMU University, Kuala Lumpur, Malaysia

**Keywords:** bioimaging, biosensing, carbon quantum dots, drug delivery, nanomaterials

## Abstract

Carbon quantum dots (CQDs) represent a rapidly developing class of fluorescent nanomaterials with increasing relevance in biomedical research and application. Their tuneable photoluminescence (PL), favourable biocompatibility, and versatile surface chemistry has supported applications in bioimaging, biosensing, and therapeutic strategies. Advances in top-down, bottom-up, and green synthesis routes have improved control over emission profiles, heteroatom doping, and surface functionalisation. Recent work has begun to elucidate how synthesis conditions and surface states govern biological interactions, intracellular transport, and subcellular localisation. This review provides an updated, mechanistic evaluation of these developments, with particular emphasis on how defined structural attributes influence antimicrobial activity, organelle-specific targeting, and integrated imaging-therapy platforms. Despite these advances, significant challenges continue to hinder clinical translation. These include variability in synthesis protocols, inconsistent batch-to-batch reproducibility, and insufficient data on long-term toxicity and biodistribution. The absence of standardised characterisation frameworks and clear regulatory pathways further complicate translational progress. Through critically linking synthesis strategies to surface chemistry and biological behaviour, this review depicts key design considerations necessary for advancing CQDs toward clinical application in next-generation nanomedicine.

## Introduction

Nanostructured carbon allotropes, including fullerenes, carbon nanotubes (CNTs), and graphene, have transformed materials science while paving novel possibilities in nanotechnology and bioengineering [1]. Carbon dots (CDs) represent a broad class of photoluminescent, nanoscale carbon-based nanomaterials, among these, carbon quantum dots (CQDs), a subset of CDs, have attracted a lot of interest due to their exceptional optical and electronic properties [2]. CQDs were first discovered in 2004 via the purification of single-walled carbon nanotubes (SWCNTs) [2]. The earliest report by Xu et al. [3], described the isolation of a highly fluorescent fraction from oxidised CNTs, thereby establishing the foundation for ensuing research into CQDs [3].

Subsequently, Sun et al. [4] provided a more definitive characterisation of CQDs by highlighting several key optical properties. The authors demonstrated that the brightness of the CQDs can be quantified through measurable quantum yield (QY) and remains stable following prolonged and repeated light exposure. In contrast to other conventional fluorescent nanoparticles that tend to exhibit intermittent photoluminescence under microscopic observation, CDs display more continuous and stable emission, attributed to surface energy trap states, which become highly emissive following effective surface passivation [4]. Collectively, CDs and CQDs are now recognised as a heterogeneous group of nanoparticles unified by their strong photoluminescence and nanometric scale [2].

At the nanoscale, quantum effects become prominent, resulting in optical and electronic behaviours that differ markedly from their bulk counterparts [2]. CQDs, often described as semiconductor “artificial atoms,” exhibit distinct size-dependent properties that govern their emission characteristics [5]. Upon photoexcitation, electrons within CQDs transition from a ground state to an excited state and subsequently emit fluorescence as they return to equilibrium. The emission wavelength is inversely correlated with particle size, with smaller CQDs (approximately 2-3 nm) typically producing blue or green fluorescence, while larger examples (approximately 5-6 nm) emit orange or red light as a consequence of reduced band gaps [5, 6]. This tuneable photoluminescence, along with excitation wavelength-dependent fluorescence, underscores the potential of CQDs in optical sensing and bioimaging.

Conventional semiconductor quantum dots (SQDs), which are commonly composed of heavy metals such as cadmium or mercury, have been widely associated with cytotoxicity due to metal ion leaching in biological environments [7]. In contrast, carbon quantum dots (CQDs) are generally reported to exhibit lower toxicity, largely because they lack heavy metal cores [8]. However, CQDs are not inherently non-toxic, as their biological effects are strongly influenced by physicochemical parameters including concentration, surface charge, particle size, and surface functional groups [9]. A study demonstrated that positively charged or highly concentrated CQDs may induce dose-dependent cytotoxicity [10]. This highlights the lack of consensus regarding a universally safe exposure threshold. In this context, surface functionalisation and chemical engineering of CQDs have emerged as critical strategies to enhance biocompatibility and mitigate potential adverse biological effects [11].

A closer examination of the literature indicates that CQD-associated cytotoxicity at higher concentrations is not universal but varies considerably across experiments models and conditions [12]. Qiang et al. [13] reported that such cytotoxic effects are primarily driven by excessive production of reactive oxygen species (ROS) [13]. They further demonstrated that exposure to high concentrations of CQDs can disrupt extracellular osmotic valence, causing cellular swelling and morphological deformation. Furthermore, CQDs at high doses were shown to interact strongly with the cell surface membrane, promoting lipid peroxidation and compromising membrane integrity. This compromised membrane integrity, facilitates CQD internalisation, where intracellular accumulation may exacerbate oxidative stress and contribute to DNA damage [14].

CQDs have gained increasing relevance in biomedical science due to their distinctive optical and physiochemical properties [2]. Their strong fluorescence, especially in the red and near-infrared regions, renders them well suited for bioimaging and diagnostic applications. CQDs have been shown to interact favourably with a wide range of human proteins, expanding opportunities for the rational design of nanomedicine-based platforms. Combined with their relatively simple, economical synthesis, and their ability to form functional polymer composites, CQDs have emerged as highly tuneable nanomaterials for biochemical, biological, and biomedical applications [2].

Common methods for synthesising CQDs include electrochemical, hydrothermal, microwave-assisted, ultrasound-assisted, oxidation, and reduction approaches [12]. However, many of these techniques involve stringent reaction conditions and long synthesis times, prompting interest in alternative routes such as pyrolysis. Pyrolysis offers a simpler, more cost-effective method that avoids high-pressure systems and expensive instrumentation, and has demonstrated advantages over conventional hydrothermal synthesis, which typically requires prolonged high-temperature and high-pressure conditions with lower yields [12].

Beyond the synthesis route, the optical performance of CQDs is strongly governed by particle size and surface chemistry [2]. Since their initial discovery during SWCNTs purification in 2004, numerous synthetic strategies have produced CQDs with diverse surface functional groups [2, 3]. As a result, surface modification and passivation have become central strategies for optimising CQD properties, as functional groups and heteroatom doping or co-doping can effectively tune absorption and photoluminescence behaviour, helping to overcome intrinsic limitations associated with CQD synthesis [2]. Despite improvements in synthesis efficiency, many studies continue to prioritise production methods over systematic optimisation of surface chemistry, which remains a key determinant of CQD optical performance and consistency.

Although CQDs demonstrate considerable potential, their long-term toxicity, *in vivo* behaviour, and complex interactions within biological systems remain incompletely understood, presenting challenges regarding safe and effective clinical translation. This review therefore examines how various synthesis methods and surface-engineering strategies influence the biomedical performance and associated applications of CQDs, while critically addressing the key challenges related to toxicity, standardisation, scalability, and clinical transition.

## Engineering Carbon Quantum Dots: Surface Functionalisation, Doping, and Their Physiochemical and Optical Properties

**FIGURE 1:**
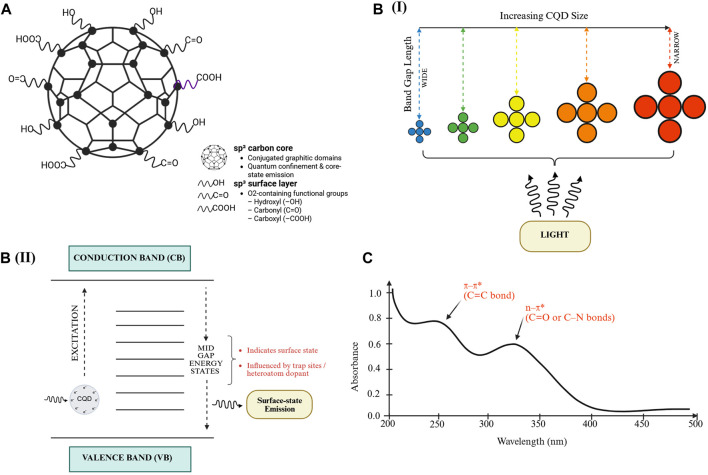
Structural, optical, and emission characteristics of carbon quantum dots (CQDs). **(A)** Schematic illustration of the general structural features of CQDs, **(B)** Optical properties of CQDs: **(I)** schematic illustration of emission colour shifts influenced by variations in particle size and band-gap energy; **(II)** steady-state absorption profile and energy band structure of CQDs. Adapted from Mohanaraman and Chidambaram (Heliyon, 2024), available at https://pmc.ncbi.nlm.nih.gov/articles/PMC11365325/, used under CC BY-NC 4.0. **(C)** UV–Vis absorption spectrum indicating π−π* (C=C) and n−π* (C=O or C−N) transitions. Adapted from Kamal, Hong, and Ju (Biosensors, 2025), available at https://www.mdpi.com/2079-6374/15/2/99, used under CC BY 4.0.

CQDs are zero-dimensional nanomaterials with characteristic diameters typically below 10 nm, primarily composed of carbon, hydrogen, and oxygen, exhibiting quasi-spherical morphology and hybridised amorphous–crystalline cores. Their structure comprises a conjugated sp^2^ carbon core and an sp^3^-hybridized surface enriched with oxygen-containing groups such as hydroxyl, carbonyl, and carboxyl moieties [15–18] (Figure 1A). These structural characteristics produce strong photoluminescence (PL) and unique electronic behaviour, distinct from graphene quantum dots (GQDs) or nanodots [15, 16, 19]. The essential defining features of CQDs are nanoscale confinement, tuneable emission arising from intrinsic or surface-related states, and versatile surface chemistry, influencing stability, solubility, and charge distribution [19–21].

The PL mechanisms of CQDs are classified into core-state and surface-state emissions. Core-state emission results from quantum confinement in sp^2^ domains, where decreasing particle size leads to blue-shifted emission due to wider band gaps, while larger conjugated domains yield red shifts [15, 19, 20] (Figure 1BI). Surface-state emission, in contrast, originates from trap sites or heteroatom dopants that introduce mid-gap states, generating emission highly sensitive to pH, solvent polarity, and oxidation level [22–24] (Figure 1BII).

For instance, CQDs derived from *Actinidia deliciosa* showed pH-dependent PL with fluorescence quenching in basic environments [23], while silk-CQD composites exhibited blue–green tuneable emissions depending on aggregation [22]. Heterogeneous surface states also explain excitation-dependent emission, where shorter excitation wavelengths activate smaller sp^2^ clusters (blue emission), and longer wavelengths excite defect states (red-shifted emission) [17, 18, 21].

Optical characterisation using UV–Vis spectroscopy typically shows π–π* transitions at 220–280 nm (C=C bonds) and n–π* transitions at 280–350 nm (C=O or C–N bonds) [19, 21, 24] (Figure 1C). Absorption tails in the visible region further indicate the role of surface states [25, 26]. QY reflects the efficiency of light emission, defined as the ratio of photons emitted to photons absorbed, and in CQDs, it can reach up to 65% depending on factors such as synthesis method, surface passivation, and dopant incorporation [24, 25].

Solvent-mediated synthesis has achieved narrow-bandwidth multicolour emissions and high QY values suitable for white light-emitting diodes [25]. Nitrogen and sulphur dopants enhance QY by increasing radiative recombination efficiency, while fluorine or phosphorus influence electron transfer and emission stability [19, 21, 27]. CQDs generally exhibit nanosecond-scale fluorescence lifetimes and remarkable photostability, making them suitable for bioimaging and optoelectronic devices [17, 20, 26].

Surface chemistry dictates much of the optical and physicochemical behaviour of CQDs. Hydrophilic groups such as hydroxyl, carboxyl, and carbonyl moieties enhance aqueous solubility and colloidal stability, while amino or thiol functionalities improve PL by enhancing electron delocalisation [27, 28]. Building upon this, heteroatom doping, involving elements such as nitrogen (N), sulphur (S), phosphorus (P), and boron (B), tailors the surface states and structures of CQDs, thereby modulating their optical and electrical properties [6, 27–30].

N-doping introduces electron-donating amino groups and sp^2^ C-N domains that enhance exciton recombination in CQDs, resulting in higher PL yields and red-shifted emissions [30–32]. S-doping introduces emissive trap states and surface functionalities that increase radiative recombination and fluorescence intensity [29, 32]. The photoexcitation from these doping effects promotes the formation of ROS [30, 32].

P- and B- doping alters the charge distribution and carbon core, modifying the energy levels and improving the flow of electrical charges and ROS yield [29, 30, 32]. Metal-doped or hybrid semiconductor, which introduces ions like Mn, Cu, or ZN, further influences electron transitions, induces magnetic properties, and enhances ROS generation for photodynamic and therapeutic applications [29, 32]. Co-doping (e.g., N/S or B/N/S) provides synergistic emission control and ROS modulation, allowing tailored applications, such as N, S co-doping for high ROS generation in phototherapy or N, P doping for red and near-infrared emission in deep-tissue imaging [19, 30–32]. Surface charge modifications, evaluated via zeta potential, determine colloidal stability and biomolecular conjugation [21, 26].

The surface functionalisation of CQDs involves both covalent and non-covalent strategies to shape surface properties and functionalisation. Covalent functionalisation forms stable bonds via amidation, esterification, or silylation, ensuring durable conjugation for drugs, proteins, or imaging agents, whereas non-covalent approaches rely on π–π stacking, electrostatic interactions, adsorption, or chelation, ensuring reversible binding, well-suited for stimuli-responsive delivery [6, 18, 33]. Introducing or modifying amino, carboxyl, and hydroxyl groups enhances solubility, biocompatibility, fluorescence stability, and payload control [6, 33]. Furthermore, stable conjugation and stealth coatings, like polyethylene glycol (PEG), allow for reduced immune clearance and prolonged circulation [18, 33].

**FIGURE 2:**
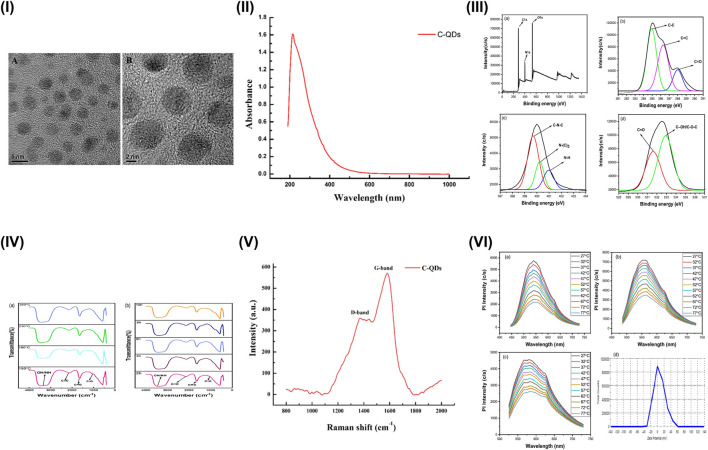
Characterisation of carbon quantum dots (CQDs). **(I)** Transmission electron microscopy (TEM) images of synthesised CQDs: **(A)** Low-resolution and **(B)** high-resolution images showing crystalline lattice fringes; **(II)** Ultraviolet-visible spectroscopy (UV-Vis) absorption spectrum of CQDs; **(III)** X-ray photoelectron spectroscopy (XPS) analysis comprising: **(a)** survey spectrum and high-resolution spectra of **(b)** C 1s, **(c)** N 1s, and **(d)** O 1s; **(IV)** Fourier transform infrared spectroscopy (FTIR) spectra of CQDs synthesised under different **(a)** temperatures and **(b)** reaction times; **(V)** Raman spectrum (785 nm excitation) showing the D band (1,371 cm^−1^), and G band (1,582 cm^−1^); (VI) Temperature-dependent photoluminescence (PL) intensity at excitation wavelengths of **(a)** 435 nm, **(b)** 475 nm, **(c)** 515 nm, and **(d)** zeta-potential (mV). **(I,II,V)** are adapted from Dager *et al.* (Scientific Reports, 2019), available at: https://www.nature.com/articles/s41598-019-50397-5, used under CC BY 4.0. **(III,IV,VI)**, are adapted from Kumar *et al.* (Scientific Reports, 2022), available at: https://pmc.ncbi.nlm.nih.gov/articles/PMC9797560/, used under CC BY 4.0. The figure panels were redrawn and adapted by the authors based on the original works.

Comprehensive characterisation confirms these relationships. Transmission electron microscopy (TEM) (Figure 2) and atomic force microscopy (AFM) assess particle size and morphology (1–10 nm), while X-ray diffraction (XRD) reveals partial graphitisation with broad peaks near 24° [26, 34]. Fourier transform infrared spectroscopy (FTIR) identifies surface groups (carbonyl, ether, hydroxyl), and X-ray photoelectron spectroscopy (XPS) quantifies elemental states [25, 26]. Raman spectra display D (∼1,350 cm^−1^) and G (∼1,580 cm^−1^) bands, with I_D/I_G ratios which quantify the degree of structural disorder or defects in the carbon lattice [32]. Complementary optical techniques such as ultraviolet-visible (UV–Vis) spectroscopy, PL spectroscopy, and zeta potential analysis confirm surface and charge-related behaviour (Figure 2) [21, 26, 34].

Overall, the combined influence of nanoscale structure, doping, and surface passivation governs the performance of CQDs. Controlled synthesis enables high emission tunability, stability, and biocompatibility, establishing CQDs as promising candidates for applications in imaging, sensing, catalysis, and photoactivated technology. Engineering strategies like heteroatom doping and surface functionalisation refine optical behaviour, enhance ROS generation, and enable targeted functionalities, broadening the application of CQDs.

## Methods of Synthesis

A wide range of synthesis techniques have been developed for CQDs, enabling precise control over particle size and surface chemistry to tailor their physiochemical properties for specific applications. These approaches encompass top-down and bottom-up strategies, green or biomass derived synthesis routes, as well as downstream purification and scale-up processes.

### Top-Down Methods

Top-down techniques, often referred to as physical methods, are used to fragment bulk materials into nanostructured materials. Several methods include laser ablation and arc discharge, mechanical milling, electrospinning, lithography, sputtering, electrochemical exfoliation and ultrasonic assisted.

#### Laser Ablation

This method utilises high-powered laser irradiation to ablate bulk carbon targets within a controlled reactive environment containing argon and water vapour. The intense laser irradiation generates high-temperature plasma plumes with ultra-high kinetic energy, which subsequently expand and cool to establish thermodynamic equilibrium. During this process, high-energy electrons transition to lower electronic orbitals, releasing photons and dissipating thermal energy, thereby facilitating CQD formation [24]. For example, Narasimhan et al. [35, 36] synthesised CQDs by focusing nanosecond-pulse laser onto pyrolytic graphite immersed in polyethylene-water solution, achieving controlled particle sizes and PL properties after heating and evaporation steps [35]. Laser ablation is considered an environmentally friendly technique, as it enables nanoparticle production without the need for stabilising agents or additional chemical reagents. This approach is broadly applicable to a wide range of nanomaterials, including metals, carbon nanomaterials, and ceramics [37, 38].

#### Arc Discharge

Arc discharge employs a high voltage arc between carbon electrodes to generate plasma within an inert gas and liquid environment. The applied electric field facilitates the vaporisation of carbon atoms from graphite into a high-energy plasma, promoting nucleation and subsequent formation of CQDs. Xu et al*.* [3] reported that additional filtration and purification of SWCNTs yielded CQDs [24]. This process involves nitric acid oxidation, which introduced hydrophilic carboxyl groups onto the CQD surface, improving aqueous solubility and resulting in a broad particle size distribution, with a QY of approximately 1.66% under 366 nm excitation. In contrast, Mujica *et al.* (2021) [39] successfully synthesised CQDs in water, exhibiting strong PL around 406 nm [19, 24]. However, CQDs produced via arc discharge frequently contain complex impurities, posing challenges for downstream purification. Despite this limitation, this method remains a prominent top-down technique for the synthesis of CQDs and other carbon nanomaterials, with synthesis conditions strongly influencing particle size, structural features, and PL properties.

#### Mechanical Milling

Mechanical milling, or ball milling, is a mechanochemical technique capable of producing a wide range of nanophases and nanocomposites. This process employs hard milling media within rotating or vibrating mills, where repeated high-energy impacts induce deformation and fracture, leading to nanoscale material refinement. Milling parameters are crucial in determining particle size, crystallinity, phase distribution, and surface characteristics. This technique offers several advantages, including facile synthesis, rapid processing, cost-effectiveness, and environmental friendliness [38, 40].

#### Electrospinning

Electrospinning is recognised as one of the simplest top-down techniques, producing nanofibers from a diverse range of materials [37]. The process involves drawing charged threads from polymer melts or solutions, forming fibres with diameters in the nanometre range. Coaxial electrospinning represents a major breakthrough, in which the spinneret consists of two coaxial capillaries. These capillaries may contain either two viscous liquids, or a viscous liquid shell surrounding a non-viscous liquid core, forming core-shell nanoarchitectures under an applied electric field. This approach provides an effective and straightforward route for large scale synthesis and has been used to develop core-shell and hollow polymeric, organic, inorganic, and hybrid materials [38].

#### Lithography

Lithography employs beams of light or electrons and can be categorised into masked or maskless approaches. Masked lithography uses photolithography, nanoimprint lithography, and soft lithography to transfer nanopatterns across large surfaces using masks or templates. Conversely, maskless lithography enables nanopatterns without the usage of masks, this includes scanning probe lithography, focused ion beam lithography, and electron beam lithography [37, 38]. An emerging technique known as direct optical lithography eliminates the need for polymer photoresists through photochemical reactions with surface ligands or additives to modify the nanomaterial film’s solubility, thereby reducing cost and environmental impact, achieving excellent resolution across a range of photon energies and wider material applicability [41].

#### Sputtering

Sputtering is an effective technique for producing thin films of nanomaterials by bombarding solid target surfaces with energetic particles generated from plasma or gas. During this process, small atom clusters are released due to high voltage gaseous ions striking the target surface within an evacuated chamber. This method yields sputtered nanomaterial composition with low impurity levels and is more cost-effective compared to electron-beam lithography [37, 38].

#### Electrochemical Exfoliation

Electrochemical exfoliation was first discovered by Zhou *et al.* [42] in 2007 using tetra-butyl ammonium perchlorate as an electrolyte to produce blue-luminescent CQDs from multiwalled CNTs into smaller components from large carbon precursors, utilising reference electrodes. Subsequent studies by Zhao *et al.* (2008) [43] utilised graphite rods as working electrodes to prepare fluorescent carbon nanomaterials, while Zheng *et al.* (2009) [44] developed water-soluble CQDs with tuneable luminescence by employing phosphate for pH buffering. Deng *et al.* (2014) [45] synthesised CQDs from low molecular weight alcohol under environment circumstances, while Hou *et al.* (2015) [46] produced bright blue emitting CQDs through electrochemical treatment of urea and sodium citrate in deionised water. This technique is cost-effective, requires no surface passivation, and uses a simple purification process, however, remains restricted by the limited selection of molecular precursors often resulting in limited application [6, 19, 35].

#### Ultrasonic Assisted

Ultrasonic-assisted methods rely on the formation and collapse of cavitation bubbles in liquid media, generating strong hydrodynamic shear forces that fragment bulk carbon materials into nanoscale CDs. The properties of resulting CDs can be tuned by adjusting ultrasonic power, reaction time, and carbon source-to-solvent ratios. Park *et al.* (2014) [47] reported the synthesis of water-soluble CDs from carbonised food waste, exhibiting good PL, low cytotoxicity, and high photostability suitable for bioimaging. Similarly, Boruah et al. [48] utilised raw coal and coal washery rejects employing modified ultrasonic-assisted wet chemical oxidation process involving hydrogen peroxide treatment, followed by ultrasonication, neutralisation, and filtration to obtain CQDs [48, 49].

### Bottom-Up Methods

In bottom-up approaches, nanostructured particles are formed through the controlled assembly of small molecules and atoms.

#### Microwave-Assisted

This technique is environmentally friendly and cost-effective, enabling the rapid production of CQDs through uniform heating that promotes the formation of CDs [49]. Water-soluble CDs have been synthesised from eggshell membrane via one-pot microwave assisted method achieving an excellent fluorescence with QY of approximately 14% and enabling detection of Cu^2+^ and glutathione [49]. Similarly, novel fluorescent CDs were synthesised via microwave assisted hydrothermal treatment of transition-metal ions and crab shell biomass, showing stability across pH changes for drug delivery [49]. In contrast to conventional slow heating, microwave synthesis rapidly heats precursors, leading to carbonisation and surface functionalisation of CQDs, enabling shorter reaction times, lower energy consumption, and high photoluminescence quantum yield (PLQY) of up to 99% [6, 24]. Furthermore, Zhu et al. (2009) [50] demonstrated rapid, environmentally compatible, and energy-efficient microwave synthesis, nonetheless, challenges in purification and particle size uniformity persist [19].

#### Hydrothermal and Solvothermal

Hydrothermal synthesis is an environmentally friendly, non-toxic and cost-effective method which is widely employed for CQDs synthesis [41, 52]. This approach facilitates the fragmentation of epoxy moieties on graphene oxide sheets into CQDs and enables regulation of morphology and size depending on reaction conditions, however, purification issues persist [6]. In addition to traditional precursors, waste biomass has emerged as an alternative carbon source. Metho *et al.* (2014) [51] investigated water soluble fluorescent CQDs from *saccharum officinarum* juice synthesised via a plant-based hydrothermal approach, enabling selective and sensitive detection of Cu^2+^ [49]. On the other hand, Lu et al. [49, 52] produced CQDs from pomelo peel with a QY of approximately 6.9%, which was utilised for sensitive detection of Hg^2+^ at low concentrations. These CQDs had attained tuneable PL, good biocompatibility, and photostability [49]. Overall, hydrothermal synthesis remains a versatile, scalable, and environmentally friendly technique for CQD production, and is often applicable in biosensing, bioimaging, and catalysis applications [49].

#### Pyrolysis

Pyrolysis, or thermal decomposition, is a powerful technique for producing CQDs from macroscopic carbon precursors, offering advantages such as short reaction time, low cost, simple operation, solvent-free processing, and scalability [24]. The process involves key stages such as heating, degradation, dehydration, and carbonisation where organic carbon-containing substances are converted into CQDs at high temperatures, typically exceeding 430 °C facilitated by alkaline or acidic conditions, promoting carbon precursor fragmentation [19].

#### Chemical Vapour Deposition

Chemical vapour deposition is widely used to produce CQDs, allowing for precise size and property control when manipulating reaction parameters such as carbon source, hydrogen flow rate, growth time, and substrate temperature. This method involves the formation of thin films on substrates through chemical reactions of vapor-phase precursors possessing volatility, stability, purity, low cost, non-toxicity, and minimal residue produced upon decomposition. Nanomaterial morphology can be influenced by catalysts such as nickel and cobalt. For instance, Fan et al*.* [12, 53] reported the synthesis of few-layer CQDs (5–15 nm lateral size, 1–3 nm height), by generating CQDs from methane in a hydrogen-argon atmosphere, followed by brief exposure to methane gas. Overall, this method produces high quality CQDs with tuneable structural and optical properties, suited for advanced biomedical applications [37, 38, 49].

#### Sol-Gel

The sol-gel method involves liquid precursor transformation into a colloidal sol, followed by the formation of a gel network through hydrolysis and condensation reactions that form metal-hydroxo or metal-oxo polymer bridges, followed by aging, drying and calcination. Parameters such as pH, precursor nature, aging duration, hydrolysis rate, and molar ratio critically influence influencing the structural and physiochemical properties of the product. The simplicity, low processing temperature, and the ease of composite fabrication are additional advantages to this method [37, 38].

#### Soft and Hard Templating

This method is widely used for producing nanoporous materials with well-controlled morphologies. The soft-template approach is straightforward and operates under relatively mild experimental conditions. It utilises soft templates such as block copolymers, flexible organic molecules, and various surfactants, where hydrogen bonding, van der Waals forces, and electrostatic forces play key roles, forming mesoporous polymeric carbonaceous nanospheres and mesoporous nitrogen-doped graphene.

**FIGURE 3:**
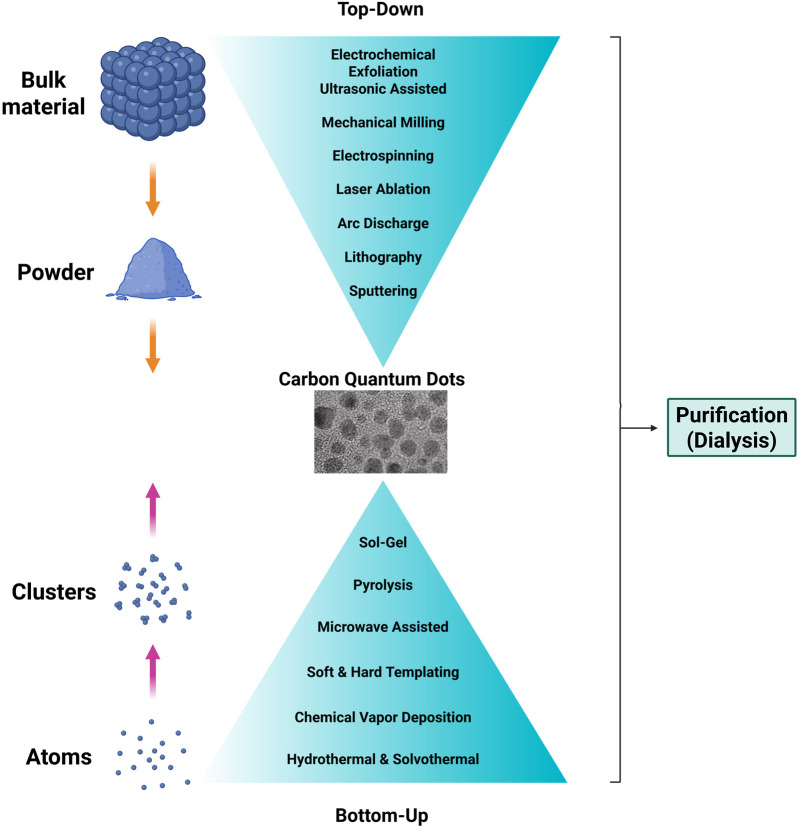
Overview of top-down and bottom-up synthesis approaches for carbon quantum dot (CQD) synthesis, illustrating representative physical and chemical synthesis routes. The central panel shows a high-resolution transmission electron microscopy (TEM) image of carbon quantum dots. Adapted from Tan *et al.* (2021), with permission.

In contrast, the hard-template method, also referred to as nano-casting, uses pre-designed solid templates with mesoporous structures. Precursors infiltrate the template and are removed to yield the desired nanostructure. The template must retain structural integrity during infiltration and be removeable without damage to the final material. This approach enables the synthesis of nanowires, three-dimensional nanostructured materials, nanorods, and metal oxides with precisely controlled morphologies [6, 19, 37]. Together, these approaches highlight the diversity of synthesis strategies available for tailoring CQD structure and morphology (Figure 3).

### Green and Biomass-Derived Precursors

CQDs synthesised from green natural precursors or biomass sources have gained significant interest, establishing this approach as a sustainable and environmentally friendly synthesis method. CQDs are mainly structured by the core structural component, carbon, which contributes to their abundant surface functional groups and their overall stability [54]. The carbon-rich precursors, such as green natural and biomass-derived materials being the most prominent contributors [55], acting as the source of carbon when incorporated with top-down or bottom-up synthesis methods. Table 1 summarises the studies reported with the utilisation of various precursor sources with different synthesis method. However, despite the abundance and accessibility of these precursors, CQDs often exhibited variability in particle size, showing the importance of further optimisation for reproducible outcomes. The variation in precursor carbon content and chemical composition also influence the variations in surface groups, structural features, and properties of CQDs synthesised.

**TABLE 1:** Carbon quantum dot (CQD) precursors and synthesis methods from biomass sources.

Category	Precursor(s)	Synthesis method	Average size of CQDs (nm)	References
Food waste-derived	Pomelo peel	Hydrothermal	1.2–8.4	[56]
	Papaya peel	Hydrothermal	4.16 ± 0.07	[57]
	Orange peel	Hydrothermal	3.5–5.5	[58]
	Avocado peel	Hydrothermal	1.5–13.5	[59]
Biomass-derived	Empty fruit bunch	Microwave	1–4	[60]
	Dried coconut leaves	Aerobic carbonisation	15.3	[61]
	Peanut shell	Hydrothermal	3.82	[62]
	Soybean polysaccharides	Hydrothermal	2.66 ± 0.69	[63]
	Barley bran	Hydrothermal method in a microwave reactor	5.5	[64]
	*Hibiscus rosa-sinensis* leaves	Microwave	12.08 ± 0.52	[65]
	Gardenia seeds biomass	Pyrolysis	0.7 ± 0.3	[12]
	*Calotropis gigantea* leaves	Reflux	<10	[66]
	*Phyllanthus emblica*	Microwave-assisted pyrolysis	2–16	[67]
	Rubber seed shells	Hummers’ approach	-	[68]
	Feijoa leaves	Microwave-assisted pyrolysis	6	[69]
Fruit-based	*Prunus armeniaca* juice	Microwave	2.6 ± 0.27	[70]
	*Bergamot pomace*	Hydrothermal	10–52.5	[71]
	*Rhus chinensis* fruit	Hydrothermal	3.37 ± 0.18	[72]
	*Citrus aurantifolia*	Hydrothermal	-	[73]
	Orange juice	Hydrothermal	12	[74]
Plant-based	*Madhuca longifolia*	Hydrothermal	5.77	[75]
	*Zanthoxylum plant*	Solvothermal	3.5	[76]
	*Syzygium aromaticum*	Carbonisation-assisted ultrasonication	3.314	[77]
	*Blumea eriantha*	Microwave-assisted technique	2.19–8.95	[78]

### Purification and Scale-Up Considerations

After synthesis with various precursors, the yield contains impure CQDs with the presence of by-products, unreacted precursors, and impurities which might interfere with the characterisation, potentially leading to experimental error [79]. This might explain the wide variation in the CQD sizes observed in certain precursors. Hence, purification techniques are essential for the isolation of pure CQDs from other unwanted products to ensure purity, while also narrowing down overall size distribution.

Dialysis is a common technique that utilises membranes with molecular weight cut-offs ranging from 0.5 to 3.5 kDa to purify CQDs. Dipcin *et al.* [80] demonstrated that three cycles of centrifugation followed by 3 days of dialysis efficiently removed impurities while preserving the surface functional groups of CQDs [80]. Similarly, González-González *et al.* [81] demonstrated that dialysis effectively eliminates residual contaminants [81], ensuring that the physicochemical properties of the CQDs can be assessed accurately without the interference of the contaminants. Raeispour *et al.* [82] also reported that the use of a dialysis membrane promotes uniform particle size distribution [82], which may otherwise vary due to batch-to-batch variation of precursors [79].

Although purification effectively removes by-products and ensures the production of CQDs with uniform size and consistent properties, achieving consistency during large-scale synthesis remains a challenge. Scaling-up often results in variation depending on the synthesis method employed, and the reaction conditions, which affects size, properties, and yield of CQDs [81]. This limitation can be addressed through continuous flow synthesis, in which precursors are continuously delivered at a fixed rate via a high-pressure pump into a heated reaction zone, followed by product collection through a condenser. This configuration enables scalable and reproducible production of CQDs in larger quantities [8].

Supajaruwong *et al.* [84] demonstrated the effectiveness of a continuous hydrothermal synthesis approach, integrating a microreactor system with a liquid chromatography pump that applies pressure [84]. The flow rate was adjusted by optimising the reaction temperature and time, obtaining the most suitable parameters to produce CDs with moderate temperature and pressure within a short reaction duration [84]. This method uses optimised reaction parameters to ensure large-scale production with reduced energy consumption, whilst minimising batch-to-batch variation compared with conventional synthesis techniques [85]. Therefore, issues related to varying size distribution and low production efficiency can be mitigated through the integration of effective purification and scalable synthesis strategies.

## Biomedical Applications

### Bioimaging and Theranostic Applications

CQDs have attracted significant interest as bioimaging agents, largely due to their tuneable PL, which facilitates real-time, minimally invasive cellular imaging through confocal and fluorescence microscopy [2]. Their UV-Vis absorption and emission properties can be tailored through surface passivation and functionalisation strategies, allowing modulation of fluorescence intensity and emission wavelength, including advantageous, red-shifted outputs for bioimaging [86]. Emission across the visible spectrum, spanning blue to red fluorescence, is generally attributed to surface defect states associated with carbon-oxygen functional groups in addition to size-dependent optical properties [87, 88]. Common approaches to modulate these emissive features involve adjusting synthesis parameters such as reaction temperature and precursor composition [89], as well as by heteroatom doping strategies that alter the electronic structure of the carbon core [90].

Beyond imaging alone, CQDs are increasingly explored as multifunctional theranostic platforms that integrate diagnostic and therapeutic capabilities. As highlighted by Daby *et al.* [91], their tuneable PL enables high contrast fluorescence imaging, however, challenges relating to *in vivo* stability, biodistribution, and long-term safety remains key barriers to clinical translation [91]. At the subcellular level, Liu *et al.* [92] demonstrated that organelle-targeting CQDs could preferentially accumulate within mitochondria, the Golgi apparatus, and the endoplasmic reticulum, illustrating how variations in precursor composition and surface functional groups can influence intracellular localisation and enhance organelle-specific imaging contrast [92].

CQDs have also been widely explored in cancer imaging. N-CQDs synthesised from lemon precursors by Tadesse *et al.* [93] exhibited bright intracellular fluorescence and good cytocompatibility in MCF-7 breast cancer cells, supporting their application in live cell imaging [93]. Similarly, auxin-derived CDs developed by Noorkhajavi *et al.* [94], also exhibited strong 1 intracellular fluorescence in murine 4T1 cancer cells, with uptake inhibition studies suggesting the involvement of receptor-mediated uptake, thereby highlighting their potential for targeted tumour visualisation [94]. Complementary work using green-derived CQDs synthesised from bread waste demonstrated efficient cellular internalisation and widespread cytoplasmic distribution in HT-29 and CT-26 colon carcinoma cells, enabling clear visualisation of subcellular features under fluorescence microscopy [95].

In addition to imaging, CQDs have shown promise as multifunctional carriers for biomolecular delivery. Several studies report the formation of stable CQD-Ct-DNA complexes capable of intracellular delivery in L929 and MCF-7 cell lines, allowing fluorescent monitoring of gene transport, moreover, suggesting potential applications as non-viral vectors for bioimaging-guided gene transport [96, 97]. More recently, red-emissive CQDs synthesised from *Echinophora tenuifolia* were shown to selectively stain non-viable cells with high fluorescent intensity and photostability, a property attributed to their negatively charged surface favouring interactions with compromised cellular membranes, sparing viable cells [98]. Taken together, these studies highlight how precise control of surface chemistry and emission properties can be leveraged to direct cellular uptake and intracellular localisation, reinforcing the potential of CQDs as versatile nanomaterials for diagnostic and therapeutic applications.

**FIGURE 4:**
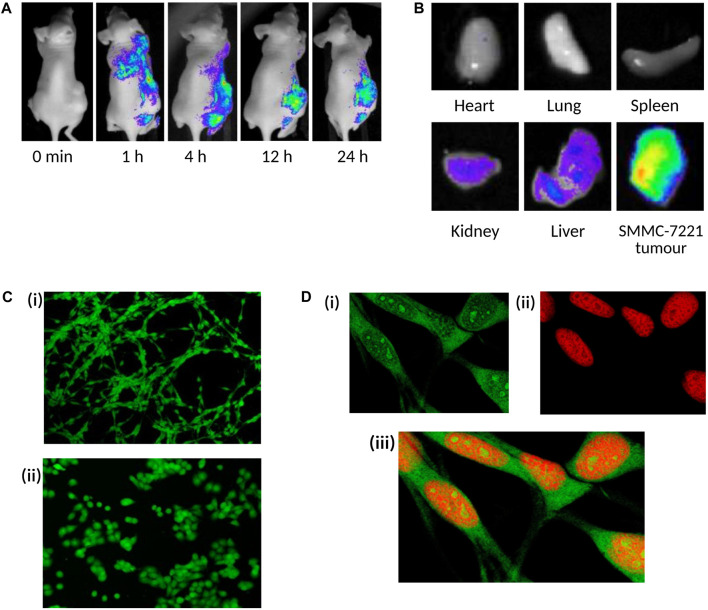
*In vivo* and *in vitro* bioimaging applications of carbon quantum dots. **(A)** Time-dependent whole-body fluorescence imaging of nude mice bearing SMMC-7221 human hepatocellular carcinoma xenograft tumours, following intravenous administration of WS-CQDs, showing progressive tumour-site fluorescence at 0 m, 1 h, 4 h, 12 h, and 24 h post-administration. **(B)** *Ex vivo* fluorescence imaging of heart, lung, spleen, kidney, liver, and tumour tissue 24 h post-administration, showing preferential tumour accumulation, alongside observed fluorescence in the kidney and liver indicating WS-CQD uptake. **(A,B)** are adapted from Zhang *et al.* (Nanomaterials, 2019), available at: https://www.mdpi.com/2079-4991/9/3/387, used under CC BY 4.0. **(C)** Confocal microscopy images of colon carcinoma cells incubated with CQD H2 for 24 h: **(i)** CT-26 and **(ii)** HT-29 cells. Green channel fluorescence (excitation peak at 431 nm and an emission peak at 540 nm) at ×20 magnification confirms efficient intracellular uptake in both cell lines. **(D)** High magnification (63x) confocal images of CT-26 cells incubated with CQD H2 for 24 h: **(i)** Green channel image showing CQD fluorescence, **(ii)** red channel showing nuclear counterstaining with propidium iodide, **(iii)** merged image demonstrating predominant cytoplasmic localisation of CQDs with limited nuclear signalling. **(C,D)** are adapted from Anpalagan *et al.* (Nanomaterials, 2023), available at: https://www.mdpi.com/2079-4991/13/14/2103, used under CC BY 4.0.

From a materials design perspective, the suitability of CQDs for bioimaging is closely linked to synthesis-dependent control over particle size, surface passivation, and heteroatom incorporation. Bottom-up synthesis approaches, particularly when combined with N or S doping, tend to produce CQDs with enhanced PL efficiency and improved emission stability. Effective surface passivation minimises non-radiative energy loss and photobleaching, which offers a rationale for the continuous fluorescence behaviour frequently reported in imaging studies. These synthesis-dependent optical features collectively govern the strong imaging performance of CQDs at the cellular and subcellular levels. Figure 4 provides representative examples of CQD bioimaging in both *in vitro* and *in vivo* models, demonstrating efficient cellular uptake and selective tumour accumulation.

### Drug and Gene Delivery

CQDs in drug and gene delivery demonstrated high effectiveness as carriers due to their non-toxic nature, biocompatibility, PL, small size and large surface area for rapid cellular uptake through binding or adsorption without affecting the drug’s activity, making them a convenient choice [2, 9]. The acidic environment of the targeted site breaks the bond between CQDs and drug, resulting in controlled release [2]. Free or unconjugated CQDs are excreted through hepatobiliary or renal system [2]. Doxorubicin a common Food and Drug Administration (FDA) licensed chemotherapy drug inhibits the growth of cancer cells by utilising enzymes from malignant cells DNA to accelerate DNA base pair intercalation [2]. For example, arginine-glycine-aspartic acid-GQDs used for drug delivery and targeted imaging purposes is an effective conjugation of doxorubicin as GQDs are able to accurately assess cellular absorption and release of doxorubicin [9]. The hydrogen bonding between GQDs and doxorubicin was shown to affect drug release. This conjugation demonstrated that its cytotoxic activity was significantly higher than of free doxorubicin when tested against U251 glioma cells [9].

In addition, Liu *et al.* (2012) [99] disclosed the encapsulation of PEG-modified nanographene oxide with the aromatic anticancer drug SN38, resulted in approximately 1000-fold greater anticancer efficacy compared with the FDA-approved treatment for colon cancer [9]. In addition, CQDs encapsulated with garlic extract within alginate beads showed enhanced surface loading, with coated CQDs achieving more than 60% higher garlic extract retention compared with uncoated alginate beads [9]. The drug released system was controlled by pH with its therapeutic effect to be found stimulating [9]. In various cancers and metabolic disorders, mitochondria play a crucial role, hence CQDs produced from precursors such as chitosan through hydrothermal synthesis can be utilised for targeted delivery to the nucleus and mitochondria, demonstrating long-term mitochondrial imaging capability [100]. Collectively, these studies suggest that the performance of CQDs in drug delivery is not determined by nanoscale size or intrinsic PL alone but is largely governed by how the particles are synthesised.

Hydrothermal and other bottom-up synthetic routes allow surface chemistry and precursor composition to be tuned during formation, enabling the incorporation of functional groups, heteroatoms, and biomimetics. These synthesis-dependent choices directly influence drug loading capacity, pH-responsive release, organelle targeting, and cellular uptake, highlighting that therapeutic efficacy arises from deliberate surface and structural engineering, rather than from the carbon core alone. In biotechnology, gene therapy plays a significant role by correcting aberrant gene expression and is regarded a long-lasting and curative therapeutic strategy [2, 9]. This technique is able to transfer genetic materials within the cells through carriers, thus attracting significant interest in non-viral nanocarriers [2]. Various classes of nanoparticles have therefore been explored to enable targeted gene transport and intracellular delivery [9].

CQDs are particularly attractive in this context due to their ease of surface functionalisation with components such as cell-penetrating peptides and targeting ligands, simultaneously enabling image-guided tracking [2, 101]. A study demonstrated the formation of stable DNA/polymer carbon dot (PCD) complexes, where DNA compaction within CDs was confirmed by agarose gel electrophoresis [9, 100]. Gene expression was visualised through green fluorescence from enhanced green fluorescent protein, while the blue fluorescence of PCDs confirmed their utility as intrinsic makers for gene delivery and bioimaging [9, 96]. Similarly, multicolour fluorescence was observed from polyethylenimine-functionalised CQDs in COS-7 cells, highlighting their multifunctional potential in gene delivery, bioimaging, and cellular labelling [2, 100]. Pandey *et al.* (2021) [102] further reported that CDs synthesised from citric acid or β-alanine, carrying a positive surface charge, enabled efficient plasmid DNA delivery into *Escherichia coli* (*E. coli)*, resulting in improved transfection efficiency and targeted gene delivery.

**FIGURE 5:**
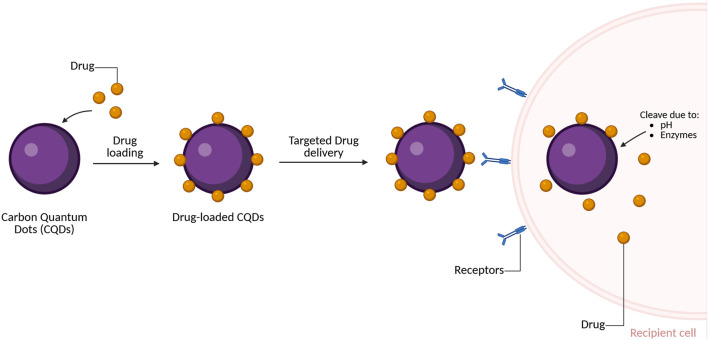
Mechanism of targeted drug delivery using carbon quantum dots (CQDs). Created in BioRender.com.

In summary, CQDs have shown extraordinary potential as carriers for both drug and gene delivery due to their physiological properties, and controlled release behaviour, combined with efficient excretion pathways, making them promising platforms for targeted therapeutic applications. The general mechanism of CQD-facilitated targeted drug delivery is illustrated in Figure 5.

### Biosensing and Diagnostics

Biosensors can be described as analytical devices that measure changes in biological processes and convert them into a readable signal, such as electrical signals [103]. Biosensors can be used in disease detection and prevention, rehabilitation, and health management [104]. CQDs are a group of fluorescent nanoparticles characterised by their tiny size, under 10 nm, with structures that vary depending on their synthesis methods [21, 105]. Moreover, the numerous functional groups on their surface (hydroxyl, amino, carboxyl) contribute to their water solubility and biocompatibility [21]. They also exhibit excellent polymerisation capabilities, allowing interactions with various biologically active, organic, and inorganic materials [21]. Surface passivation further improves their physical robustness and photoluminescent efficiency [21]. Collectively, these features make CQDs particularly suitable for integration into biosensing platforms and diagnostic systems.

Among the exploited mechanisms in CQD-based biosensing is fluorescence quenching. Fluorescence quenching refers to the loss or reduction in the fluorescence of CQDs after reacting with quenchers [24]. Quenching occurs through non-reactive decays linked to mid-gap states in the CQD band gap energy as they dissipate excitation energy as heat, preventing electron-hole recombination for the generation of fluorescence [24]. Further, photoinduced electron or energy transfer, whereby CQDs function as electron acceptors or donors [24]. For example, iron ions (Fe^3+^) have shown to strongly inhibit CQD fluorescence by accepting photoexcited electrons [24, 106]. Khan *et al.* [107] have reported a negative linear relationship between CQD fluorescence intensity ratio and Fe^3+^ concentrations [107]. These responses make them beneficial for use in highly sensitive optical biosensors for selective monitoring of metal ions.

Additionally, fluorescence of N-CQDs have shown to be selective fluorescence quenching effects upon interaction with dopamine molecules, due to the electrostatic reaction between CQD surface groups and the amino groups of dopamine molecules, showing potential for dopamine sensing in biological fluids [108]. Surface defects and aggregation are other factors which could contribute to the fluorescence quenching of CQDs due to unwanted electronic states of higher density which trap photo-excited carriers, enhancing non-radiative decays [108]. For instance, solvo-thermally synthesised yellow emissive CQDs have been used to quantify bilirubin in human urine and serum [109].

Furthermore, combining CQDs with rhodamine 6G can be used to monitor glucose levels [110], where a change in colour from blue to green indicates an increase in glucose levels and has good selectivity for glucose over the major components in human blood and can be used with serum [110]. These biosensors increase the accessibility to detect glucose using the naked eye [110]. Yu *et al.* [111] reported that combining CQDs with glucose oxidase and cellulose acetate complex sensitive film leads to high selectivity, reproducibility and anti-interference ability for real-time detection of low concentrations of glucose. By utilising the fluorescence quenching ability of the film, the biosensor can manifest a rapid fluorescence response to glucose [111].

Moreover, CQDs can act as an electrochemical biosensor, which effectively detects and measures the movement of electrons to determine the analyte [112]. CQDs act as simple immobilising compounds for the development of enzymatic biosensors due to their high surface-to-volume ratio and adaptive nature and can be enhanced using redox-active enzymes [112]. Liu et al. [113] developed an electrogenerated chemiluminescence (ECL) biosensor by combining N-CQDs with DNA to detect microRNA (miRNA)-21 [113]. Whereby an initial DNA sequenced was utilised to conjugate CQDs to aid in the hybridisation of miRNA and the assistance probe [113]. The nicking enzyme identified this complex and cleaved the DNA, releasing the miRNA to engage in more hybridisation cycles [113].

After DNA cleavage, the miRNA hybridises with a detection hairpin immobilised on a graphene oxide electrode, which produces an ECL signal that can be quantitatively measured [112, 114, 115]. Furthermore, mixing nanomaterials with CQDs could improve their properties [112, 114, 115]. Pourmadadi *et al.* [115], developed an electrochemical aptasensor to determine prostate-specific antigen (PSA) using CQDs and gold nanoparticles [115]. The interaction between the nanomaterials showed enhancement in the electrical conductivity of CQDs following the rise in the current peak of cyclic voltammetry (CV) in COQ-gold nanoparticles compared to CQDs [115]. In addition, CQDs can enhance ECL performance by promoting electron transfer and providing a stable interface for biomolecule immobilisation using TiO_2_-CQD medium, where the medium’s pH value could be altered to measure total-PSA and free-PSA separately. CV was used to examine the changes, showing a linear relationship between the concentration of the sample solution and the resistance of the electron transfer on the surface of the electrode [114].

Taken together, these biosensing studies demonstrate that CQD sensing performance is predominantly dictated by synthesis-driven control over surface chemistry and defect states rather than optical properties alone. Heteroatom doping, surface passivation, and the deliberate introduction of oxygen- and nitrogen-containing functional groups during synthesis generate reactive surface sites that govern analyte binding, electron transfer, and signal transduction. Consequently, sensitivity and selectivity across fluorescence, electrochemical, and ECL-based biosensors emerge from rational surface and defect engineering at the synthesis stage, underscoring the tight coupling between synthetic design and biosensing functionality, which is also directly extended to the detection of ROS.

ROS are reactive forms of oxygen which include superoxide anion radical, hydrogen peroxide, singlet oxygen and hydroxyl radical, they are usually produced during normal metabolism of oxygen inside the mitochondria [116]. Peroxynitrite, a kind of ROS in which its overproduction is linked to various diseases. Zhou *et al.* [117] developed a strategy to prepare CDs with various oxygen-rich surface groups that could selectively detect peroxynitrite in living cells, showing a linear chemiluminescence response and exhibiting efficient cellular uptake, shown by emission of strong green PL in cells after co-incubation. Stimulation with SIN-1 or PMA can enable real-time monitoring of both exogenous and endogenous peroxynitrite production [117]. Since CQDs possess structural and optical features similar to CDs, it is anticipated that they will exhibit comparable performance in detecting ROS.

### Photodynamic and Photothermal Therapies

Photodynamic therapy (PDT) uses specific wavelengths of light to activate photosensitisers (PSs), which can be derived from natural or synthetic compounds [103]. The photosensitisers produce ROS that kill tumour cells. However, oxygen dependence of PDT results in limitation of its efficacy against hypoxic tumours. Further, conventional PSs have low stability, poor solubility and potential damage to neighbouring tissues [103]. To overcome these limitations CQDs can be encapsulated with hematoporphyrin (HP) (HP-CQDs) using HP monomer as a precursor [118]. The product retained optical and chemical properties of HP with significant improvement in water solubility [118]. HP-CQDs can generate ROS effectively under red light and has enhanced effectiveness in PDTC against MCF-7 human breast cancer cells [118]. In comparison with HP alone, HP-CQDs have higher phototoxicity and lower dark toxicity, allowing them to be more effective in targeting cancer cells [118].

Various CQD-based systems have shown potential anticancer effects. Li *et al.* [119] synthesised CQDs from tender ginger juice which inhibited tumour growth in mice within 14 days [119], whereas He *et al.* [120] developed Diketopyrrolopyrrole-based CDs which are highly photostable and capable of strong cellular uptake and tumour suppression at low concentrations [120]. CDs containing porphyrin are minuscule in size, have good water solubility and are photostable [121]. They generate singlet oxygen under irradiation which induces cell apoptosis, inhibiting the growth of hepatoma. Beack *et al.* [122], synthesised CQD-chlorine e6-hyaluronate conjugates that generated singlet oxygen at higher efficacy compared to free chlorine e6, leading to complete suppression of B16F10 murine melanoma cells following laser irradiation [122]. Through these observations, we can conclude that different CQD compositions cam be used as potent anticancer agents upon visible light irradiation.

Other than the generation of ROS from CQDs, the antibacterial mechanism of CQDs have been recorded such as DNA binding, membrane destabilisation, physical and mechanical damage and inhibition of bacterial metabolism. Thus, preventing bacteria from developing resistance [121]. Positively charged or N-CQDs interact electrostatically with the negatively charged bacterial membranes (lipopolysaccharides, lipoteichoic acids), leading to enhanced bacterial adhesion and antibacterial activity [123]. N-CQDs synthesised via a one-step chemical route affected the cell structure of *Staphylococcus aureus* (*S. aureus*) and methicillin-resistant *S. aureus* (MRSA) but were less effective against *Escherichia coli* (*E. coli*) [124].

CQDs can also be encapsulated into polymer films and hydrogels to form potent photodynamic antibacterial surfaces [121]. These components demonstrate strong antibacterial activity against *S. aureus, E.coli* and *Klebsiella pneumoniae* under blue light irradiation. Gamma ray irradiation of these components induced change in morphology and chemical composition which contributed to increased antibacterial activity towards *S. aureus* and *E. coli* [125]*.* Moreover, CQD thin films exhibited bactericidal effects against MRSA and *Pseudomonas aeruginosa* [126].

In summary, these findings highlight CQDs as potential light-activated antimicrobial agents, effective at preventing and treating infections. Their modifiable surface properties, biocompatibility and ROS-generating capabilities make them promising candidates for photoactivated disinfection, antibacterial coatings, and wound-healing applications, especially in environments where antibiotic resistance remains a growing concern [121].

Furthermore, CQDs have demonstrated significant potential as multifunctional agents in cancer therapy acting both as a nanocarrier as well as a photosensitiser for PDT [127]. For instance, carbon quantum dots clathrates loaded with methotrexate, conjugated with folic acid receptors, enabled targeted drug delivery to tumour cells. Upon near infrared laser irradiation (1064 nm), the formation of ROS and increased release of medication into cancer cells were observed.

Shahshahanipour *et al.* [128], developed a method of synthesising CQDs using *Lawsonia inermis* (henna) via hydrothermal procedure, where the CQDs demonstrated excellent stability and fluorescence characteristics, without surface functionalisation. Their use as biocompatible fluorescent probes for selective methotrexate detection in human serum via Förster resonance energy transfer [128]. Another study demonstrated CQDs loaded with doxorubicin or combined with multifunctional platforms (*i.e.*, FeN@CQDs with folic acid and riboflavin, or SWCNT-PEG-Fe_3_O_4_-CQDs-doxorubicin conjugates) that demonstrate combined chemotherapy and PDT effects with increased specificity toward cancer cells [127], further elucidating the theranostic potential of CQDs, where their fluorescence allows imaging and tracking, while their capacity to deliver drugs and generate ROS under light exposure enhances their therapeutic efficacy.

Cumulatively, these studies indicate that the therapeutic performance of CQDs in photodynamic and photothermal applications is fundamentally governed by synthesis-driven modulation of photophysical properties rather than by the carbon core alone. Heteroatom co-doping, defect engineering, and hybridisation with photosensitisers or metallic components enhance light absorption, charge separation, and ROS or heat generation, directly influencing treatment efficacy and tissue penetration. However, increasing structural and compositional complexity also introduces challenges related to batch-to-batch reproducibility, long-term biosafety, and regulatory assessment. Consequently, balancing phototherapeutic performance with synthetic simplicity and reproducibility remains a critical consideration for advancing CQD-based systems toward clinical translation.

### Antimicrobial Applications of Carbon Quantum Dots

Emerging evidence indicates that CQDs can exert antiviral effects across diverse viral systems, although translational relevance varies by model. In a plant virology model, Farooq *et al.* [129] demonstrated that cysteine-functionalised CQDs were shown to reduce begomoviral titres and symptom severity in *Nicotiana bethamiana*, concurrently modulating immunity-related gene expression, including pathways associated with endocytosis and sphingolipid signalling [129]. Although this work highlights antiviral potential in a botanical host, it is important to note that its applicability to mammalian antiviral systems is limited. In contrast, coal-derived CQDs exhibited potent inhibition of SARS-CoV-2 replication *in vitro*, achieving more than 95% reduction in viral growth at half-maximal inhibitory concentration (IC_50_) of approximately 5.47 μg/mL, with minimal cytotoxicity in normal cell lines, indicating genuine antiviral activity in a human-relevant viral system [123].

Similarly, Chen *et al.* [130] reported that curcumin-derived CQDs demonstrated potent antiviral activity against the Japanese encephalitis virus by selectively binding to its surface envelope protein. Through binding key domains involved in host cell attachment and membrane fusion, these CQDs effectively disrupted early-stage viral infection, resulting in a marked reduction in viral entry and replication, suggesting that surface functionalisation can confer targeted disruption of viral-host interactions [130]. Consistent with these findings, recent reviews have identified recurring antiviral mechanisms among carbon-dot-based nanomaterials, including viral surface protein interference and redox-mediated viral inactivation, however, they also emphasis the lack of standardised synthesis, limited *in vivo* validation, and incomplete toxicological characterisation, which collectively constraint clinical translation [131].

CQDs have also demonstrated considerable promise as antibacterial agents, particularly when engineered to enhance redox activity or integrate into composite nanostructures. Chen *et al.* [132] reported that CQD-ZnO nanocomposites containing 7–14 wt% CQDs exhibited significantly enhanced antibacterial effects against *Streptococcus mutans, Enterococcus faecalis, and E. coli* compared with ZnO alone. This effect was attributed to increased ROS generation and bacterial membrane disruption [132]. Similarly, Shen *et al.* [133] synthesised N-CQDs from *Solanum nigrum* extract and reported minimum inhibitory concentrations of approximately 1.1–1.2 mg/mL against *S. aureus* and *E. coli* [133]. Mechanistic analyses revealed that bacterial inactivation was associated to hydroxyl radical generation, and electrostatic interactions between positively charged N-CQDs and negatively charged bacterial membranes, leading to morphological changes and cell wall damage [133].

At a broader level, a review by Collins *et al.* [134] highlighted the antimicrobial versatility of CQDs across foodborne and clinically relevant pathogens, noting common mechanisms such as oxidative stress induction, biofilm inhibition, and interference with bacterial metabolic pathways [134]. In addition, Ghacham *et al.* [135] reported that green-synthesised CQD-silver nanocomposites exhibited broad spectrum antibacterial activity alongside accelerated wound-healing, attributed to the synergistic effects of silver ion-mediated membrane disruption and CQD-driven oxidative stress [135]. Collectively, these studies support the versatile antibacterial potential of CQDs through various mechanisms, however, most evidence remains derived from *in vitro* systems, with limited *in vivo* validation and safety profiling, highlighting the need for further evaluation prior to clinical translation.

Beyond their antiviral and antibacterial applications, CQDs have also been explored for antifungal applications, although evidence remains comparatively limited. Belal *et al.* [136] reported that N-CQDs significantly reduced fungal counts in both *in vitro* assays and an *in vivo* rat model of mucormycosis, with antifungal effects attributed to ROS generation, disruption of fungal cell wall integrity, and increased membrane permeability [136]. While these findings suggest potent fungicidal activity, the study represents a preclinical investigation, and further validation in larger, clinically relevant models are required to establish therapeutic relevance. In relation, Slewa *et al.* [137] demonstrated that lemon-and onion-derived CQDs incorporated into biodegradable packaging films effectively inhibited the growth of *Rhizopus spp., Penicillium spp., Candida albicans, Aspergillus spp.,* and *Botrytis cinerea*, highlighting the function of CQDs in surface-associated antifungal applications in addition to their therapeutic uses [137].

Overall, the available evidence supports CQDs as versatile antimicrobial nanomaterials capable of interfering with viral entry, bacterial membrane integrity, and fungal viability, largely through surface-dependent interactions and oxidative mechanisms. However, further work is required to standardise synthesis approaches, define safety profiles, and validate efficacy in physiologically relevant models to support future clinical translation. The antiviral, antibacterial, and antifungal activities of CQDs are strongly governed by synthesis-driven modulation of surface chemistry, charge, and defect density rather than by the carbon core alone.

**FIGURE 6:**
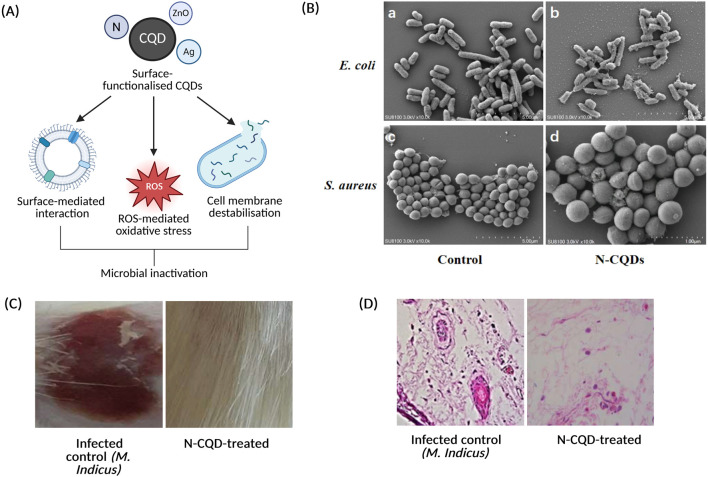
Antimicrobial applications of carbon quantum dots. **(A)** Representative schematic illustration of the proposed antimicrobial actions of surface-functionalised CQDs, highlighting surface interactions with microbial cells, ROS-mediated oxidative stress, and cell membrane destabilisation, contributing to microbial inactivation, created in https://BioRender.com. **(B)** SEM analysis of antibacterial activity: **(a)** *Escherichia coli* control **(b)** *Escherichia coli* treated with N-CQDs **(c)** *Staphylococcus aureus* control, and **(d)** *Staphylococcus aureus* treated with N-CQDs, showing morphological deformation and collapse following exposure to N-CQDs, reproduced from Shen *et al*. (Sci Rep, 2025) 15:28535. doi:10.1038/s41598-025-14383-4, licensed under Creative Commons Attribution- NonCommercial-NoDerivatives 4.0 International (CC BY-NC-ND 4.0) http://creativecommons.org/licenses/by-nc-nd/4.0/. **(C,D)** *In vivo* wound healing evaluation, comprising **(C)** macroscopic assessment of infected wounds, showing representative images of the infected control (*M. indicus*) and N-CQD-treated groups, demonstrating enhanced wound closure following treatment; and **(D)** corresponding histological analysis, where infected control tissue (*M. indicus*) exhibits inflammatory cell infiltration and structural disruption, whereas N-CQD-treated tissue shows reduced inflammation and improved tissue morphology, adapted from Belal *et al*. (Artificial Cells, Nanomedicine & Biotechnology, 2024) available at: https://www.tandfonline.com/doi/full/10.1080/21691401.2024.2318212, used under CC BY 4.0.

Heteroatom doping, surface functionalisation, and composite formation enhance electrostatic interactions with microbial membranes, promote ROS generation, and enable targeted disruption of viral surface proteins or microbial cell walls. However, while increasing structural complexity can amplify antimicrobial potency, it also raises challenges related to reproducibility, biosafety, and the lack of standardised toxicological evaluation. Consequently, translating CQD-based antimicrobial systems toward clinical or applied use will require careful balancing of antimicrobial efficacy with controlled synthesis, rigorous safety profiling, and validation in physiologically relevant *in vivo* models. Figure 6 summarises the proposed antimicrobial mechanisms of CQDs, alongside representative *in vitro* antibacterial, and *in vivo* wound-healing evidence.

### Delivery of Carbon Quantum Dots Through the Blood-Brain Barrier

The blood-brain barrier (BBB) represents a major translational obstacle in the treatment of neurological diseases and remains one of the greatest challenges in the development of effective neurotherapeutics [138]. Formed by tightly apposed endothelial cells supported by pericytes and astrocytic end-feet, the BBB restricts the entry of most small molecules, biologics, and nearly all gene-editing constructs [139]. While essential for maintaining cerebral homeostasis, this barrier concurrently limits pharmacological interventions, often resulting in sub-therapeutic drug concentrations [140]. In response to these limitations, CQDs have emerged as promising candidates for central nervous system (CNS) delivery, owing to their ultrasmall size, tuneable surface chemistry, and potential to engage endogenous transport mechanisms [141].

Seven *et al.* [142] reported that glucose-derived CDs successfully crossed the BBB transporting small molecular cargo in both murine and zebrafish models, providing evidence of *in vivo* BBB permeability [142]. While glucose transporter-mediated uptake was experimentally validated in budding yeast, the specific mechanism underlying BBB translocation in vertebrates remains inferred rather than directly demonstrated. The authors noted that glucose transporter expression is upregulated following neurological injury, suggesting potentially enhanced delivery potential [142]. However, transporter involvement in physiologically relevant BBB models remains to be mechanistically confirmed, warranting further validation in mammalian systems. Recent reviews indicate that suitably engineered CQDs can traverse the BBB via receptor-mediated endocytosis and adsorptive-mediated transcytosis, with surface charge and amphiphilicity influencing endothelial interactions and uptake efficiency, although the relative contributions of these pathways are not yet well defined [143]. Consequently, careful surface engineering is critical in achieving reliable and translatable CNS delivery.

Protein misfolding and aggregation underlie major neurodegenerative diseases such as Alzheimer’s (AD), Parkinson’s, and Huntington’s disease, further characterised by neuronal loss, mitochondrial dysfunction, toxic fibril formation, and oxidative stress. Guerrero *et al.* [144] demonstrated that sodium-citrate CQDs could prevent hen egg-white lysosome (HEWL) from forming mature amyloid fibrils, a common model of amyloidogenic protein aggregation [144]. In addition, these CQDs disaggregated mature fibrils with low cytotoxicity, indicating their potential for both prophylactic and therapeutic intervention in protein-misfolding disorders [144]. Similarly, Mukherjee *et al.* [145] showed that microwave-synthesised CQDs significantly reduced HEWL amyloidogenesis by delaying nucleation, inhibiting b-sheet conversion, and disrupting protofibril elongation, ultimately yielding fewer and shorter aggregates with reduced cytotoxicity [145].

While these studies highlight the neuroprotective potential of CQDs in controlled assays, HEWL is a model protein with limited structural homology to disease-relevant aggregates, and the *in vitro* assays do not fully reflect neuronal environments. Therefore, translation to human neurodegenerative diseases remain uncertain. Lim *et al.* [146] further reported that curcumin-derived CQDs inhibited amyloid-b fibril aggregation and the associated oxidative stress in AD models via interactions with b-sheet domains, preventing peptide self-assembly, and concurrent curcumin-mediated free-radical suppression [146]. Overall, while these findings demonstrate promising neuroprotective effects, the pharmacokinetics, long-term biodistribution, and potential off-target toxicity of CQDs remain largely unexplored, highlighting the need for more relevant studies prior to clinical application.

Crossing the BBB is particularly advantageous in malignancies such as glioblastoma multiforme (GBM), where diffuse infiltration and chemoresistance hinder surgical and pharmacological management. GBM constitutes the most malignant subtype of glioma and the most prevalent primary brain tumour in adults, accounting for 45.2% of cases [147]. Yan *et al.* [145] developed paclitaxel-derived carbon quantum dots (PTX-CDs) which penetrated an *in vitro* BBB model, achieving a reported permeability of 19.8%. In an orthotopic glioblastoma mouse model, PTX-CDs accumulated within intracranial tumours and exerted antitumour effects through ROS-mediated oxidative stress [145]. Mechanistic analysis using endocytosis pathway inhibitors indicated that PTX-CDs predominantly traverse the BBB via macropinocytosis, highlighting the involvement of energy-dependent vesicular uptake mechanisms in BBB translocation [148].

At the cellular level, PTX-CDs localised to mitochondria, disrupted mitochondrial membrane potential, and induced oxidative stress-mediated cytotoxicity [148]. However, while this dual mechanism of drug delivery and ROS-generation enhances antitumour efficacy, the potential for off-target oxidative damage to healthy brain cells and associated neurotoxicity was not comprehensively evaluated, which may in-turn limit translational interpretation. Similarly, Algarra *et al.* [149] showed that 2-acrylamido-2-methylpropanesulfonic-acid-derived CDs, effectively delivered riluzole to GBM cells, significantly reducing cell viability while exhibiting negligible intrinsic toxicity, thereby highlighting their biocompatibility and therapeutic stability as nanocarriers [149]. Complementing this, Hettiarachchi *et al.* [150] reported that transferrin-conjugated CDs co-loaded with temozolomide and epirubicin produced markedly enhanced cytotoxicity across multiple GBM cell lines compared to single-drug formulations, illustrating the advantage of combined targeting and multi-drug delivery strategies [150]. The ultrasmall particle size of around 3.5 nm following conjugation further supported BBB uptake and intracellular delivery [150]. Collectively, these studies reinforce the versatility of CQDs, however comprehensive *in vivo* assessments of BBB transport, biodistribution, and long-term neurotoxicity are still required to substantiate clinical translational potential.

Accumulating evidence underscores the emerging neuroprotective potential of CQDs across diverse experimental models. Zhang *et al.* [151] reported that CDs derived from *Crinis carbonisatus* significantly reduced infarct volume whilst improving neurological function in a rat middle cerebral artery occlusion/reperfusion model. The effects were attributed to decreased BBB permeability, suppression of pro-inflammatory cytokines (TNF-a and IL-6), and modulation of excitatory and inhibitory neurotransmitter signalling [151]. Extending these findings, Mosalam *et al.* [152] demonstrated that hyaluronic acid-modified verapamil-loaded CQDs, exhibited enhanced neuronal uptake and neuroprotective efficacy in an *in vitro* amyloid-induced neurotoxicity model, suggesting transporter-mediated BBB translocation and enhanced intracellular drug distribution [152].

Complementary mechanistic insight is provided by recent work on free-radical-scavenging-nanoparticles, including CQDs, which highlights their ability to attenuate neuroinflammation and oxidative stress through ROS scavenging and suppression of lipid peroxidation, thereby positioning CQDs as both therapeutic nanocarriers and active redox modulators [153]. Collectively, these studies suggest that CQDs can be rationally engineered to traverse the BBB, deliver neuroactive agents, and directly modulate inflammatory and oxidative pathways. However, the predominance of *in vitro* and acute *in vivo* models highlights the need for systematic evaluation of long-term safety, biodistribution, and translational relevance in clinically relevant neurological settings [151–153]. A summary of various CQD systems engineered for BBB transport and neurotherapeutic applications is provided in Table 2.

**TABLE 2:** Blood-brain barrier uptake and neurotherapeutic applications of carbon quantum dots (CQDs).

System/Surface functionalisation	Key mechanistic feature	Neurotherapeutic applications	References
Glucose-derived CDs	Transporter-mediated BBB uptake (glucose transporter pathways)	CNS delivery without targeting ligands (mice and zebrafish models)	[142]
Sodium-citrate CQDs	Inhibit amyloid fibrillation; disaggregate oligomers	Anti-amyloid activity; potential therapeutic use in AD	[144]
Microwave-synthesised CQDs	Interfere with nucleation and beta-sheet conversion	Inhibit amyloidogenesis; neuroprotection	[145]
Curcumin-derived CQDs	ROS scavenging	Ab inhibition and antioxidative neuroprotection in AD models	[146]
PTX-CDs	Macropinocytosis-driven BBB uptake	GBM therapy with *in vivo* tumour inhibition	[148]
AMPS-derived CDs	High drug-loading stability	Riluzole delivery to GBM cells with reduced toxicity	[149]
Transferrin-conjugated CDs	Receptor mediated BBB uptake; ultrasmall size (∼3.5 nm)	Targeted combination therapy with temozolomide and epirubicin in GBM cell lines	[150]
*Crinis carbonisatus*-derived CDs	Anti-inflammatory	Stroke neuroprotection in rat MCAO/reperfusion models	[151]
Hyaluronic acid-verapamil CQDs	Transporter-mediated uptake; enhanced delivery	Improved neuronal drug distribution and neuroprotective efficacy	[152]

Abbreviations: CDs, carbon dots; CQDs, carbon quantum dots; PTX, paclitaxel; AMPS, 2-Acrylamido-2-methylpropane sulfonic acid; BBB; blood-brain barrier; ROS, reactive oxygen species; CNS, central nervous system; AD, Alzheimer’s disease; Ab, antibody; GBM, glioblastoma multiforme; MCAO, middle cerebral artery occlusion.

**FIGURE 7:**
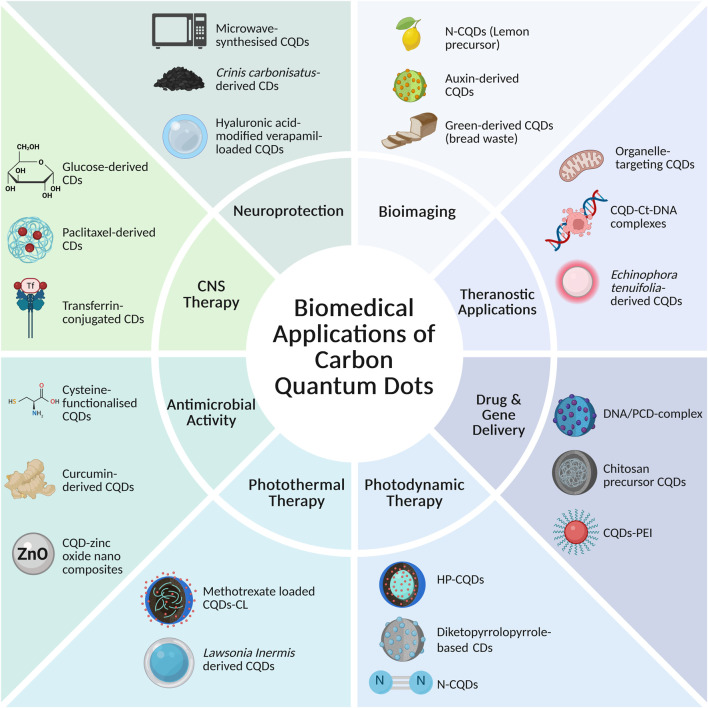
Schematic overview of representative carbon quantum dot (CQD) precursors and engineering strategies for biomedical applications. CDs, carbon dots; N-CQDs, nitrogen-doped carbon quantum dots; ct, circulating tumour; DNA, deoxyribonucleic acid; PCD, polymer carbon dot; PEI, polyethylenimine; HP, hematoporphyrin; CL, clathrate-like; CNS, central nervous system.

Across these diverse biomedical applications, ranging from bioimaging and biosensing to drug delivery, phototherapy, antimicrobial action, and neurotherapeutics, a common design principle is revealed - the biomedical behaviour of CQDs is governed primarily by precursor choice and synthesis-driven surface engineering, rather than by the carbon core itself. Differences in precursor chemistry, heteroatom doping, and surface functionalisation consistently dictate optical properties, cellular uptake, subcellular localisation, and therapeutic performance. This modularity enables CQDs to be rationally tailored toward various objectives through deliberate design choices during synthesis. To consolidate these design-function relationships across biomedical contexts, Figure 7 provides a schematic overview of representative precursors and engineering strategies discussed in this section.

## Biocompatibility, Pharmacokinetics, and Toxicity

### *In Vitro* Cytotoxicity

Recent studies have highlighted the favourable *in vitro* biocompatibility and functional versatility of plant-derived CQDs across various biomedical applications. Yalshetti *et al.* [65] reported that CQDs synthesised from *Hibiscus rosa-sinensis* exhibited moderate cytotoxicity in L929 fibroblasts with approximately 76% cell viability at 100 μg/mL, while showing minimal toxicity in HaCaT keratinocytes, supporting their selective safety profile for nanotherapeutic research [65]. Similarly, *Spinacia oleracea*-derived CQDs maintained high viability in human mesenchymal stem cells at lower concentrations, and displayed dose-dependent reductions at higher exposure concentrations, indicating their suitability for bioimaging and stem cell-related applications [154]. Nitrogen-doped CQDs (N-CQDs) synthesised from castor seeds demonstrated negligible cytotoxicity in WI-38 lung fibroblasts across a wide concentration range, highlighting their safety profile in prolonged cellular studies and pH-sensing applications [155].

In addition to favourable biocompatibility, several plant-derived CQDs have demonstrated functional activity in disease-relevant cellular models. For instance, artichoke leaf–derived CQDs showed effective dose-dependent anticancer activity in MCF-7 cells, with an IC_50_ of 96.5 μg/mL, while retaining strong fluorescence properties, thereby demonstrating dual utility in bioimaging and cytotoxicity-based research [156]. In addition, Kamble *et al.* [157] synthesised red-fluorescent CQDs (R-CQDs) from *Lawsonia inermis*, which displayed minimal toxicity in both fibroblast and cancer cell lines and exhibited notable antioxidant activity, supporting their application as safe bioimaging probes [157].

Additionally, CQD-based platforms have also been adapted for intracellular sensing, illustrated by cobalt-conjugated N-CQDs, which functioned as a non-toxic fluorescent sensors for dichloroacetic acid in HuH-7 cells, enabling real-time pharmacological monitoring without compromising cell viability [158]. However, it is important to note that the majority of these investigations employ *in vitro* models, and predominantly plant-derived CQDs, which, although advantageous for initial biocompatibility screening, may not accurately represent their behaviour in the human body.

Other studies have highlighted the capacity of these CQDs to be tailored for targeted biomedical applications. Boron-doped CQDs (B-CQDs), namely Formulation 1 (F1) and Formulation 2 (F2), preserved greater than 86% viability across multiple cell lines, demonstrating organ-specific targeting capabilities relevant to bioimaging and drug delivery strategies [159]. Finally, *Syzygium malaccense*-derived CQDs (SM-CQDs) exhibited weak cytotoxicity in 3T3-L1 adipocytes, with an IC_50_ of 444.5 μg/mL, while enhancing glucose uptake, reflecting their promise as biocompatible nanomaterials for metabolic and therapeutic applications [160]. Collectively, these findings indicate that carefully engineered CQDs offer low *in vitro* toxicity while offering broad functional potential in biomedical research and applications. Thus, while cellular studies indicate low toxicity and functional potential, further *in vivo* studies are required to assess distribution, metabolism, and clearance, as well as their long-term effects on organs and immune response (Table 3).

**TABLE 3:** Summary of carbon quantum dot (CQD)*in vitro* cytotoxicity in mammalian cell lines.

CQD source	Surface chemistry/modification	Cell line(s)	Concentration range (µg/mL)	Cytotoxicity evaluation		Additional assays	Ref
				Assay	Cell viability/IC_50_		
*Hibiscus rosa-sinensis*	Negatively charged surface; -OH, -COOH	L929, HaCaT	L929: 100–500 μg/mL; HaCaT: 1–1,000 μg/mL	MTT	L929: 76% (100 μg/mL), 28% (500 μg/mL); HaCaT: 60% (1,000 μg/mL) vs. control	Annexin V/Propidium iodide (PI) apoptosis assay, haemolysis, protein denaturation, ELISA, COX-2 inhibition	[65]
*Spinacia oleracea*	-OH, C=O, C-H	hBMSCs	1–1,000 μg/mL	CCK-8	1 μg/mL: 1.07 × (PBS, 1 day); 10 μg/mL: no change (PBS, 3 days); 100 μg/mL: reduced viability (PBS, 3 days); 1,000 μg/mL: 35% reduction (PBS, 3 days)	Annexin V/PI apoptosis assay, osteogenic differentiation	[154]
N-doped CQDs (castor seed)	N-doped; -OH, -COOH	WI-38	3.9–500 μg/mL	MTT	No significant loss of viability	Anti-microbial (MIC test)	[155]
Artichoke-derived CQDs	-OH, -COOH, C=O, C=C, C-H, C-O-C, -C-N-C	MCF-7	7.8–1,000 μg/mL	MTT	Reduced at 1,000 μg/mL; IC_50_ = 96.5 ± 5.3 μg/mL	Anti-microbial (MIC test)	[156]
R-CQDs	-NH_2_, -COOH, -OH	L929, MCF-7	1 μg/mL stock; 0.5–1 µL increments	MTT	L929: 80.64% increase; MCF-7: 75.80% increase (max dose)	DPPH, KMnO_4_ reduction, haemolysis, angiogenesis assay	[157]
N-CQDs/N-CQDs–Co	-OH, -NH/-OH, C-H, C=N, C-O, amide N-H, amide C=O; N-doped; Co^2+^ interaction	HuH7	10 μg/mL (N-CQDs); 10 + 10 μg/mL (N-CQDs/Co)	MTT	No substantial impact on viability	​	[158]
B-CQDs (F1 & F2)	-OH, C-O-C, C-H, C=O	MCF-7, A549, h-TERT	31.25–4,000 μg/mL	MTT	F1: 89.32% (31.25 μg/mL); F2: 86.15% (31.25 μg/mL) vs. untreated	Annexin V/PI (apoptosis assay)	[159]
SM*-*CQDs	-OH, C=O, C-O-C	3T3-L1	1–1,000 μg/mL	MTT	90.33% (1 μg/mL); 89.70% (10 μg/mL); 78.61% (100 μg/mL); 48.02% (500 μg/mL); 31.22% (1,000 μg/mL)	Glucose uptake, α-amylase, α-glucosidase inhibition	[160]

Abbreviations: CQDs, carbon quantum dots; N-CQDs, nitrogen-doped CQDs; R-CQDs, red fluorescent CQDs; B-CQDs, boron-doped carbon quantum dots; SM-CQDs, *Syzygium malaccense*-derived CQDs; CCK-8, cell counting kit-8; DPPH, 2,2-diphenyl-1-picrylhydrazyl; ELISA, enzyme-linked immunosorbent assay; MTT, 3-(4,5-dimethylthiazol-2-yl)-2,5-diphenyltetrazolium bromide; hBMSCs, human bone marrow-derived mesenchymal stem cells; PBS, phosphate-buffered saline; COX-2, cyclooxygenase-2; MIC, minimum inhibitory concentration.

### *In Vivo* Biodistribution and Pharmacokinetics

CQDs demonstrate organ-specific accumulation influenced by particle size, surface charge, and administration route. In murine models, *Spinacia oleracea*-derived CQDs showed safe distribution at concentrations up to 100 μg/mL with no histological organ damage, whereas higher doses elicited mild liver lymphocyte infiltration [154]. Additionally, B-CQDs administered intravenously localised in kidneys and lymph nodes, intraperitoneally in intestines, liver, heart, and spinal cord, and orally in liver, kidneys, and lymphatic tissue, with peak tissue accumulation occurring at 30 min, and rapid systemic clearance within 2 hours [159].

Furthermore, cobalt-conjugated N-CQDs demonstrated efficient penetration into neuronal and non-neuronal tissues in zebrafish, a well-established non-mammalian vertebrate model, without eliciting detectable apoptosis or oxidative stress [157]. Smaller (<6 nm), hydrophilic, negatively charged CQDs generally undergo efficient renal clearance, whereas larger or positively charged particles show partial retention in the reticuloendothelial system (RES), especially in the liver and spleen [161, 163]. Tumour-targeting CQDs preferentially accumulate in neoplastic tissues, minimising off-target organ deposition (Table 4).

**TABLE 4:** *In vivo* biodistribution and clearance of carbon quantum dots (CQDs) in mammalian models.

CQD type	Model	Dose/Route	Organ accumulation	Clearance	Reference(s)
*Spinacia oleracea*	C57BL/6 mice	≤100 μg/mL oral	No damage to major organs; high dose: mild liver infiltration	Renal	[154]
Cobalt-conjugated N-CQDs	Zebrafish (non-mammalian)	10–20 μg/mL	Neuronal and non-neuronal tissues	Efficient; no toxicity	[158]
B-CQDs	Swiss albino mice	IV/IP/Oral 5–10 mg/kg	Kidney, liver, heart, intestines, lymph nodes	Renal; partial RES	[159]
Negatively charged CDs	Mice	1–10 mg/kg	Liver, spleen	Renal (smaller particles under 6 nm)	[161,162]
Positively charged CDs	Mice	1–10 mg/kg	Liver, kidney, some RES retention	Partial renal; long-term persistence possible	[162,163]

Abbreviations: CQDs, carbon quantum dots; B-CQDs, boron-doped CQDs; CDs, carbon dots; RES, reticuloendothelial system.

Neurotoxicity evaluations, although limited, indicate that CQDs are generally non-neurotoxic at standard doses. Henriquez *et al.* [164] reported that citric acid-derived CQDs effectively mitigated oxidative stress in SH-SY5Y neuroblastoma cells, while also preserving dopaminergic neuronal integrity in paraquat-exposed models [164]. In a related line of investigation, Raut *et al.* and Güven *et al.* [158, 159] demonstrated that B-CQDs and cobalt-conjugated N-CQDs elicited no detectable oxidative stress, apoptotic signalling, or microglial activation within neuronal tissues [158, 159]. Extending this safety profile beyond the CNS, Chen *et al.* and Mishra *et al.* [163, 165] have reported that off-target toxicity in peripheral organs remained minimal, although they emphasised that excessively high doses or positively charged CQDs may still provoke ROS formation, mitochondrial dysfunction, or the activation of inflammatory pathways [163, 165].

Across these studies, CQD uptake, cytotoxicity, and organ accumulation are strongly influenced by surface chemistry and dose. Positively charged CQDs exhibit enhanced cellular uptake, but greater organ retention and potential oxidative stress, whereas negatively charged or neutral particles show lower tissue retention and minimal mitochondrial or oxidative perturbation. Most CQDs are rapidly cleared renally, limiting long-term retention, although doped or larger particles may persist in liver, spleen, or tumour tissue [161, 166].

The consistent evaluation framework includes *in vitro* cytotoxicity assays including MTT, CCK-8, and Trypan Blue, apoptosis and necrosis analysis, ROS quantification, haemolysis testing, and fluorescence imaging. *In vivo* assessments employ histopathology, serum biochemistry, organ fluorescence imaging, and multi-organ biodistribution analysis. Oxidative stress markers (HO-1, ROS), inflammatory markers (NF-κB), mitochondrial integrity, and tight junction proteins provide sensitive indicators of sub-lethal toxicity, guiding safe biomedical translation [162, 166].

Collectively, CQDs exhibit high biocompatibility, low cytotoxicity, and predictable biodistribution when engineered with the appropriate size, surface charge, and doping. Dose-dependence, surface chemistry, and renal clearance or RES clearance critically influence both therapeutic efficacy and potential off-target effects. Positively charged and metal-doped CQDs show heightened oxidative and inflammatory responses, while negatively charged or hydrophilic CQDs remain generally safe. Future investigations should address chronic exposure, long-term retention, neurotoxicity, and standardised reporting to ensure safe clinical translation.

## Standards, Reproducibility, and Regulatory Considerations

A major barrier to the biomedical and clinical translation of CQDs lies in the absence of standardised characterisation and reporting frameworks. Since different laboratories utilise a wide range of synthesis methods, including template-based calcination, hydrothermal and solvothermal reactions, resulting CQDs often display inconsistencies in size, charge, and fluorescence behaviour [167]. Even fundamental optical phenomena such as up-conversion and down-conversion fluorescence remain incompletely understood and inconsistently measured, limiting meaningful comparisons across studies [167]. Techniques such as XRD and dynamic light scattering provide information on crystallinity and hydrodynamic size, but are often unreliable for amorphous or highly polydisperse CQD formulations, further highlighting the need for harmonised, cross-validated analytical protocols [167].

The growing emphasis on green synthesis introduces additional layers of variability. While the use of natural precursors reduces reliance on toxic metals and aligns with sustainability goals, the diverse heterogenous phytochemical compositions in plant extracts can significantly influence CQD surface chemistry, charge distribution, and photoluminescent properties [168, 169]. Such variability creates significant challenges for reproducibility particularly as few studies systematically evaluate how source material variability affects CQD composition and performance. Consequently, the implementation of standardised purification procedures and compositional profiling is essential before CQDs synthesised via green routes can be compared meaningfully between studies [168, 169].

Manufacturing scalability presents a further obstacle to clinical translation. CQDs are typically produced at milligram- to gram-scale, and attempts to scale production to kilogram levels introduces batch-to-batch variability in morphology, optical output, and surface functionality [85, 170]. Key synthesis parameters including precursor concentration, reaction temperature, and time heavily influences product consistency [170]. In response, continuous-flow microreactor synthesis has been proposed to overcome these issues, offering improved mixing, uniform heating, and real-time monitoring to enhance reproducibility [85, 170]. In parallel, solvent selection has been shown to modulate emission behaviour, with comparative studies of aqueous and alcoholic extracts reporting wavelength shifts linked to differences in dielectric constant and phenolic content [170].

Effective purification remains equally critical, as unreacted precursors and luminescent impurities can obscure or distort the intrinsic optical properties of CQDs, especially those synthesised from complex biological materials [79]. Although strategies such as continuous-flow synthesis show promise, further systematic validation across multiple CQD systems and production scales is required to establish their general applicability.

Regulatory oversight adds yet another layer of complexity. At present, regulatory agencies like the FDA and the European Commission evaluate CQDs on a case-by-case basis, irrespective of a specific regulatory procedure as these nanomaterials do not fit neatly within existing classifications for pharmaceuticals, biologics, or medical devices [172]. To facilitate eventual regulatory approval, comprehensive analytical characterisation conducted in accordance with Good Laboratory Practice (GLP) standards is essential [173]. This includes transparent reporting of synthesis conditions, purification steps, surface chemistry, size and charge data, PLQY, and biological testing protocols, alongside *in vitro* and *in vivo* dosing regimens with validated positive and negative controls [174].

Without standardised characterisation and transparent documentation, CQDs will continue to face challenges in reproducibility, regulatory uncertainty, and limited clinical translation progress. This persistent disconnect between laboratory-scale development and clinical requirements likely contributes to the slow progression of CQDs toward approved biomedical applications.

## Future Perspectives and Emerging Directions

Although CQDs have demonstrated broad biological applicability and favourable biocompatibility in conventional *in vitro* and animal studies, these models do not fully capture the complexity of human physiology [86]. Consequently, there is an increasing emphasis on incorporating advanced, human-relevant models, such as organoids and organ-on-a-chip (OoC) systems to generate more predictive and translational data for future research into CQDs [86, 175]. When human cells are integrated into microfluidic platforms that mimic key physiological parameters, including oxygen gradients, fluid flow, and multicellular tissue interactions, these systems provide improved translational relevance than conventional models [175, 176].

Notably, controlled fluid flow introduces sustained perfusion and physiologically relevant shear forces, both of which modulate cellular behaviour and allow for a more accurate assessment of nanoparticle transport, distribution and cellular uptake than static *in vitro* models [175, 176]. Furthermore, the integration of biosensors and induced pluripotent stem cells enables real-time monitoring and patient-specific insight, which are crucial in nanoparticle safety assessment and the development of personalised nanomedicine strategies [175]. However, the widespread adoption of organoid and OoC models remains constrained by technical complexity, reproducibility and standardisation challenges, scalability issues, and evolving regulatory frameworks [177]. Addressing these barriers are essential before such systems can be routinely implemented for comprehensive evaluation and translation.

Beyond biological evaluation, successful clinical translation of CQDs requires strict adherence to Good Manufacturing Practice (GMP) standards to ensure reproducibility, quality control, and patient safety throughout production [178]. Large-scale CQD synthesis is often hindered by batch-to-batch variability, impurity profiles, and contamination risks, emphasising the need for standardised, GMP-compliant synthesis protocols and robust quality control frameworks [178]. Despite the availability of numerous well-established laboratory synthesis approaches, truly scalable methods capable of delivering consistent particle size, high purity, and reliable reproducibility, remain limited.

Consequently, many promising CQD formulations may fail to advance beyond laboratory scale-development, restricting their translational potential. Furthermore, regulatory approval requires case-by-case evaluation by authorities such as the FDA and European Medicines Agency, emphasising the need for manufacturing consistency and comprehensive safety evaluations [178]. In this context, green synthesis strategies using renewable, low-toxicity precursors offer a complementary route towards sustainable scale-up synthesis, while preserving the desired optical and quantum properties critical for biomedical performance [179].

From a materials design perspective, the development of hybrid and multifunctional CQD systems represent promising direction in enhancing translational relevance [91]. Integration with metals or polymers enable for precise modulation of optical, magnetic, and surface properties, improving effectiveness in imaging, biosensing, and targeted drug delivery applications [180, 181]. For example, Guan *et al.* [180] demonstrated that the incorporation of CQDs with gold, iron oxide, nickel, or PEG polymers, enhances charge transfer and optical responsiveness, supporting multimodal diagnostic functionality [180].

Similarly, doped and polymer-based CQDs, including nitrogen-, copper-, and poly (lactic-co-glycolic acid)-doped systems, have shown improved stability, synergistic fluorescence, theranostic capabilities [91, 182]. Hybrid CQD-gold and CQD-iron oxide nanostructures have allowed for photothermal therapy, fluorescence imaging, and MRI-guided drug delivery, further illustrating their potential as multifunctional theranostic platforms [181]. However, increasing structural and compositional complexity introduces challenges related to long-term safety evaluation, regulatory approval, and reproducible manufacturing, highlighting the need for rigorous validation frameworks [183].

In parallel, artificial intelligence (AI) and machine learning approaches are being increasingly leveraged to accelerate CQD optimisation, simultaneously reducing experimental burden. By correlating synthesis parameters, such as precursor composition, temperature, and ligand type, with physiochemical and biological outcomes, computational models enables predictive control over particle size, QY, fluorescence behaviour, and toxicity profiles [184–186]. Neural network-based models have demonstrated improvements in PLQY and emission predictability, particularly when integrated with microfluidic synthesis platforms that enhance reproducibility [186]. Nonetheless, the effectiveness of AI-driven optimisation remains constrained by the limited availability of high-quality, standardised datasets that reflect physiologically relevant conditions, limiting direct translational applicability [187].

Encouragingly, recent developments have begun to address several key translational challenges associated with CQDs [188]. For instance, large amino acid-mimicking glutathione-CQDs exhibit high physiological stability, reduced protein corona formation due to steric amino acid shielding, and excellent water solubility, enhancing their suitability for intravenous administration while mitigating unintended biological interactions [188]. These materials also demonstrate low haemolytic activity, favourable *in vivo* biosafety, and localised tumour accumulation via L-type amino acid transporter 1-mediated uptake, enabling improved drug delivery with reduced systemic toxicity [188].

Importantly, their straightforward and scalable synthesis demonstrates how rational materials engineering can directly contribute to overcoming translational barriers and advancing the clinical development of CQDs [103, 188]. In parallel, increasing emphasis on regulated and reproducible synthesis strategies aims to reduce batch-to-batch variability, and improve biological evaluation dependability [91, 103]. Complementary *in vivo* studies, including zebrafish models, are also providing valuable insight into CQD biodistribution, clearance pathways, and long-term safety profiles across different development stages [103, 189]. Collective findings from these studies underscore persistent translational challenges, including size heterogeneity, rapid renal clearance, and limited long-term toxicological data, highlighting the need for improved targeting, surface modification, and synthesis control to support clinical translation [91, 190].

Despite these advances, several critical challenges continue to impede the practical clinical translation of CQDs [103]. Foremost among these is the incomplete understanding of long-term toxicity, biodistribution, and clearance, which raises ongoing concerns regarding *in vivo* safety [103, 174]. Furthermore, variability in physicochemical properties arising from differences in synthesis methodologies, and the lack of standardised characterisation protocols complicates reproducibility and regulatory evaluation [103].

Although CQDs have demonstrated substantial promise in imaging, sensing and drug delivery, advancement beyond preclinical research remains restricted by the scarcity of comprehensive toxicological data and large-scale validation, and addressing these challenges will require close interdisciplinary collaboration across chemistry, materials science, bioengineering, pharmacology, and clinical medicine to establish reliable synthesis methods, robust testing, and safety frameworks aligned with regulatory guidelines [103, 174]. Ultimately, while CQDs represent a highly versatile and multifunctional nanoplatform, their translational success will depend on systematic validation, standardised manufacturing, and stronger integration between fundamental research and clinical application [103].

## Conclusion

CQDs represent a rapidly advancing class of nanomaterials in biomedical research, offering multifunctional capabilities across imaging, sensing, and therapeutic applications. Their tuneable optical properties, specific surface-engineering strategies, and favourable biocompatibility support a broad range of experimental uses, including intracellular imaging, simultaneous imaging and therapy, antiviral and antimicrobial strategies, and emerging neuroprotective applications. This versatility reflects the capacity of CQDs to be rationally engineered for various biological contexts, addressing limitations associated with conventional approaches. Despite this progress, translation beyond preclinical systems remains limited by insufficient standardisation of synthesis and purification protocols, significant batch-to-batch variability, and incomplete understanding of pharmacokinetics, long-term toxicity and biodistribution. Overcoming these challenges require coordinated interdisciplinary efforts, implementation of GMP-compliant production, and clearer regulatory frameworks. Innovations such as continuous-flow synthesis, OoC models, and machine-learning-driven strategies are required to improve reproducibility, scalability, and predictive safety assessments. As synthesis and regulatory frameworks continue to mature, CQDs are positioned to progress from experimental fluorescent probes toward clinically relevant theranostic platforms. Their integration with hybrid nanostructures, targeted ligands, and stimuli-responsive systems define the next-generation of personalised nanomedicine. Collectively, the evidence suggests that with rigorous standardisation and comprehensive biological evaluation, CQDs hold substantial potential to contribute to future diagnostic and therapeutic technologies in nanomedicine.

## Glossary

AD: Alzheimer’s disease
AI: Artificial Intelligence
AFM: atomic force microscopy
BBB: blood-brain barrier
B-CQDs: boron-doped carbon quantum dots
CCK-8: cell counting kit-8
CDs: carbon dots
CNTs: carbon nanotubes
CNS: central nervous system
CQDs: carbon quantum dots
CV: cyclic voltammetry
DLS: dynamic light scattering
DPPH: 2,2-diphenyl-1-picrylhydrazyl
ECL: electrochemiluminescence
ELISA: enzyme-linked immunosorbent assay
FDA: Food and Drug Administration
FTIR: Fourier transform infrared spectroscopy
GBM: glioblastoma multiforme
GMP: good manufacturing practice
GQDs: graphene quantum dots
hBMSCs: human bone marrow-derived mesenchymal stem cells
HEWL: hen egg-white lysosome
HP: hematoporphyrin
IC50: half-maximal inhibitory concentration
MIC: minimum inhibitory concentration
miRNA: microRNA
MRI: magnetic resonance imaging
MTT: 3-(4,5-dimethylthiazol-2-yl)-2,5-diphenyltetrazolium bromide
N-CQDs: nitrogen-doped carbon quantum dots
OoC: organ-on-a-chip
PBS: phosphate buffered saline
PCDs: polymer carbon dots
PDT: photodynamic therapy
PEG: polyethylene glycol
PL: photoluminescence
PLQY: photoluminescence quantum yield
PSs: photosensitisers
PSA: prostate-specific antigen
PTX-CDs: paclitaxel-derived carbon dots
QY: quantum yield
R-CQDs: red fluorescent carbon quantum dots
RES: reticuloendothelial system
ROS: reactive oxygen species
SWCNTs: single-walled carbon nanotubes
TEM: transmission electron microscopy
UV-Vis: ultraviolet-visible spectroscopy
WS-CQDs: wheat straw-derived carbon quantum dots
XPS: X-ray photoelectron spectroscopy
XRD: X-ray diffraction
